# Multi-omic profiling reveals metabolic vulnerabilities in enzalutamide resistant prostate cancer

**DOI:** 10.1038/s41420-026-03332-3

**Published:** 2026-09-09

**Authors:** Olivia Lee, Gio Fidelito, Qing Zhao, Bingshou Liu, Hyungwon Choi, Renea A. Taylor, Matthew J. Watt

**Affiliations:** 1 Department of Anatomy and Physiology, The University of Melbourne, Melbourne, VIC Australia; 2 Department of Medicine, National University of Singapore, Singapore, Singapore; 3 Cardiovascular Metabolic Disease Translational Research Programme, Yong Loo Lin School of Medicine, National University of Singapore, Singapore, Singapore; 4 Department of Physiology, Monash Biomedicine Discovery Institute, Monash University, Melbourne, VIC Australia; 5 Cancer Immunology Program, Cancer Research Division, Peter MacCallum Cancer Centre, Melbourne, VIC Australia; 6 Sir Peter MacCallum Department of Oncology, The University of Melbourne, Melbourne, VIC Australia; 7 Cabrini Institute, Cabrini Health, Melbourne, VIC Australia

**Keywords:** Prostate cancer, Proteomics, Cell death, Lipidomics, Metabolomics

## Abstract

Resistance to the androgen receptor inhibitor enzalutamide (ENZ) is a major barrier to durable control of advanced prostate cancer. Given the central role of metabolic reprogramming in tumour survival under therapeutic stress, we investigated whether ENZ resistance is associated with adaptive metabolic rewiring that promotes stress tolerance and evasion of cell death. Multi-omic profiling of ENZ-sensitive (C4-2B and V16D) and ENZ-resistant (MR49F) cells revealed extensive metabolic reprogramming, with lipid metabolism emerging as a dominant axis. ENZ-resistant cells displayed depletion of long-chain polyunsaturated fatty acids (PUFAs) and increased resistance to lipid peroxidation and ferroptosis. Restoring PUFA availability or activating lipid regulatory pathways via treatment with a liver X receptor (LXR) agonist re-sensitised resistant cells to ferroptotic cell death. In parallel, nitrogen metabolism was altered in ENZ-resistant cells, including increased carbamoyl phosphate synthetase 1 (CPS1) and resistance to ammonium exposure. Treatment with the CPS1 inhibitor H3B-120 reduced proliferation of MR49F cells and increased their sensitivity to ammonium stress. Together, these findings identify coordinated lipid and nitrogen remodelling as targetable metabolic adaptations that support resistance to cellular stress and death in ENZ-resistant prostate cancer cells.

**Figure:**
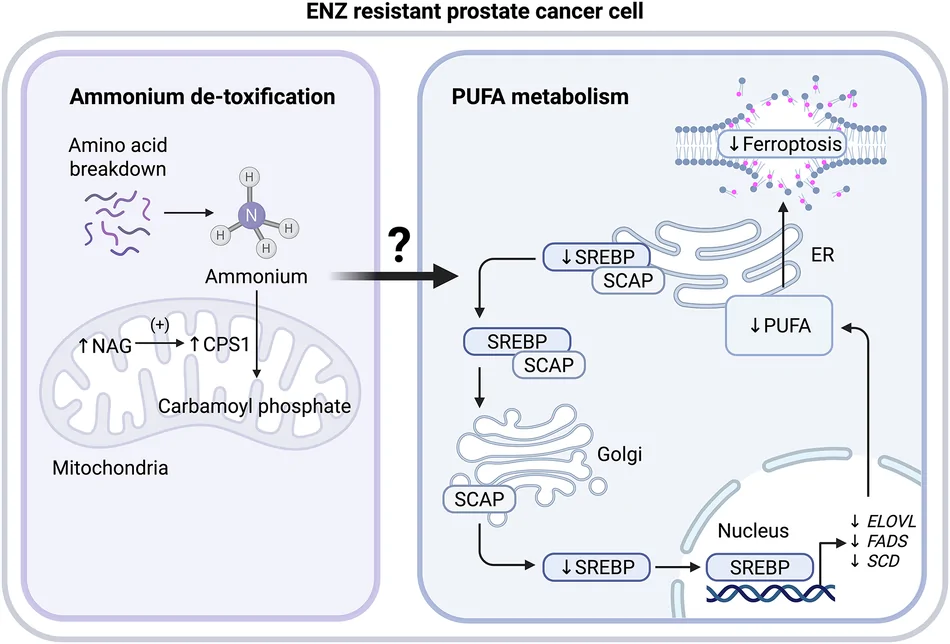

## Introduction

Prostate cancer is a heterogeneous disease comprising multiple subtypes that differ in their cellular origin, genomic alterations, and clinical pathology [1]. While localised prostate cancer is well managed with surgery and radiotherapy, ~20% of prostate tumours metastasise to other sites in the body within several years of diagnosis [2]. Metastatic prostate cancer remains incurable, with five-year survival of ~30%, underscoring a major unmet clinical need and the urgency for novel therapeutic approaches [3].

The majority of prostate tumours are driven by androgen receptor (AR) signalling and depend on androgens for growth and survival [4]. Enzalutamide (ENZ) is a potent AR antagonist that competitively binds to the ligand-binding domain of the AR to block its activation [5]. By suppressing AR-dependent signalling, ENZ is effective in reducing tumour burden by 54–78% in metastatic prostate cancer patients, prolonging patient survival and improving quality of life [6, 7]. All patients inevitably become resistant to ENZ, with tumour recurrence typically occurring 8–12 months following treatment [6–8]. Furthermore, administration and failure of anti-AR therapies, like ENZ, can promote more aggressive and therapy-resistant subtypes of prostate cancer, such as neuroendocrine prostate cancer (NEPC) [9, 10].

Although resistance to ENZ is linked to diverse genetic, transcriptomic, and epigenetic alterations of the AR, therapies that directly target AR reactivation have failed to overcome ENZ resistance [5]. These limitations underscore the importance of investigating non-canonical mechanisms of resistance, which may provide new opportunities to overcome therapy failure. One of the fundamental hallmarks that drives tumour initiation, growth and therapy resistance is the reprogramming of cellular metabolism (also known as the deregulation of cellular energetics) [11]. Proliferation is a metabolically demanding process, as cells must acquire and/or synthesise sufficient nucleotides, amino acids, and lipids required for cell division [12]. Beyond supporting proliferation, metabolic reprogramming can also influence the susceptibility of cancer cells to stress-induced cell death. For example, lipid composition and redox balance can dictate sensitivity to ferroptosis, a form of iron-dependent cell death [13], while nitrogen handling pathways influence cellular tolerance to ammonium-induced cytotoxic stress [14].

During malignant transformation, prostate epithelial cells undergo a major metabolic shift from a specialised, citrate-secreting state to a more bioenergetic, citrate-oxidising phenotype, supporting greater tricarboxylic acid (TCA) cycle activity and anabolic growth [12]. In localised disease, this is accompanied by AR-driven anabolic reprogramming, including increased de novo lipogenesis and broader rewiring of central carbon metabolism to support the biosynthetic and bioenergetic demands of tumourigenesis [15, 16]. Prostate cancer development also leads to increased uptake of exogenous lipids, which are channelled into membrane and biomass synthesis, reinforcing the highly lipogenic phenotype that is now recognised as a defining feature of prostate cancer metabolism [17]. As disease progresses to advanced and metastatic states, prostate tumours often acquire stronger glycolytic features, particularly under androgen-deprived conditions, but still require persistent TCA cycle/oxidative phosphorylation (OXPHOS) activity [18]. Advanced/metastatic prostate cancer retains the capacity to take up and utilise multiple nutrient substrates, including glutamine and fatty acids [12, 19, 20]. Only a limited number of studies have assessed metabolic reprogramming in the context of ENZ resistance. ENZ-resistant cells can increase reliance on glucose handling through heightened dependence on glucose transport and glycolytic flux, particularly in androgen-deprived contexts, creating a flexible balance between lactate-producing and oxidative states that supports survival under drug pressure [21–24]. In parallel, diversion of glucose into the pentose phosphate pathway becomes more important to generate antioxidant and biosynthetic molecules, providing a metabolic “bypass” that can sustain growth and blunt ENZ efficacy [25–27]. Altered glutamine and serine utilisation have also been shown to buffer oxidative stress in ENZ-resistant prostate cancer [28, 29]. Finally, lipid metabolism emerges as a dominant adaptive axis in ENZ resistance, including shifts towards increased de novo lipogenesis and fatty acid oxidation [24, 30, 31]. From a therapeutic perspective, pharmacological inhibition of the lipid metabolism enzyme sphingosine kinase was shown to enhance ENZ efficacy in prostate cancer models through induction of ER stress and lipogenic pathway activation [32]. These findings collectively highlight reprogrammed metabolism as a feature of ENZ resistance.

Despite growing recognition that metabolic rewiring can support survival under AR pathway blockade, how metabolism is reprogrammed in ENZ-resistant prostate cancer remains incompletely understood. Most studies have characterised metabolic changes that occur in response to acute ENZ exposure [22, 33–35], which does not necessarily reflect changes that accompany stable, fully established ENZ resistance. To date, few studies have profiled the lipidomic and metabolomic landscapes, and aligned these with proteomic changes, which limits the ability to resolve coordinated, system-level metabolic adaptations across interconnected pathways governing nutrient utilisation and associated cell functions.

To address this limitation, our study employs a combinatory multi-omics strategy centred on lipidomic, metabolomic and proteomic profiling of ENZ-sensitive and ENZ-resistant prostate cancer cells. Using this approach, we found that the cellular proteome, lipidome and polar metabolome are remodelled in ENZ-resistant prostate cancer cells. We identified polyunsaturated fatty acid synthesis and ammonium detoxification as processes that were altered in ENZ-resistant disease and tested whether targeting these pathways may provide a rationale for metabolically guided therapeutic strategies in ENZ-resistant prostate cancer.

## Results

### Radiolabelled metabolic tracing reveals reprogrammed glutamine utilisation in ENZ-resistant prostate cancer cells

**Fig. 1:**
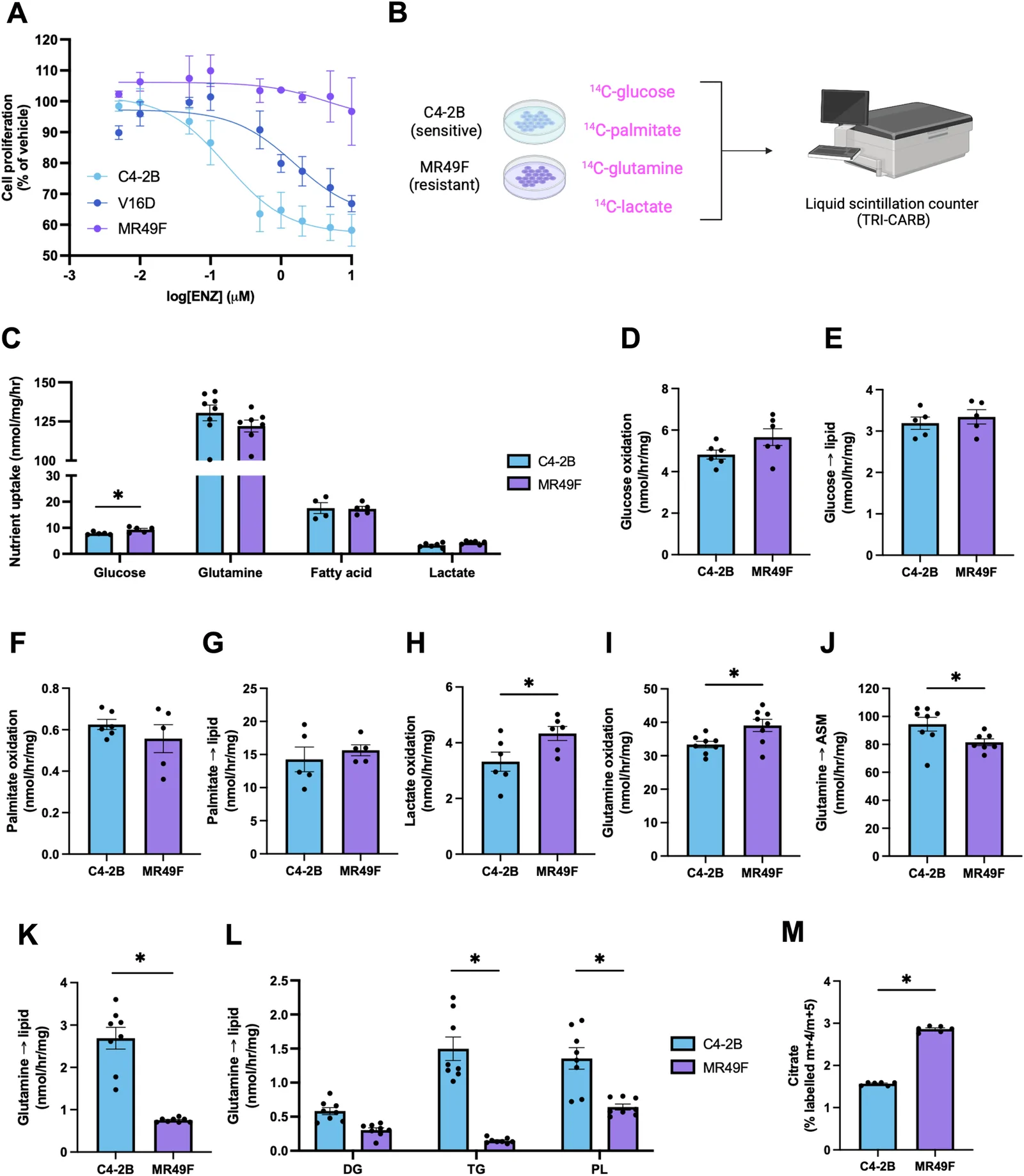
Nutrient consumption and utilisation are altered in C4-2B and MR49F prostate cancer cells. **A** Dose-response curve (non-linear fit) showing cell proliferation of C4-2B (blue, *n *= 3), V16D (dark blue, *n *= 3) and MR49F (purple, *n *= 3) cells as a percentage (%) of vehicle treatment at increasing concentrations of ENZ (0.01, 0.05, 0.1, 0.5, 1, 5 and 10 µM) following six days measured by IncuCyte SX5 live-cell imaging. **B** Schematic of experimental design tracing the metabolic fate of glucose, glutamine, fatty acids, and lactate in C4-2B and MR49F cells using radiolabelled isotopic tracing. Created in BioRender.com. **C** Rate of uptake of glucose (*n *= 5), glutamine (*n *= 7), palmitate (*n *= 5), and lactate (*n *= 6) in C4-2B and MR49F cells (nmol/mg/hr). **D** Rate of glucose oxidation (*n *= 6), and **E** incorporation into lipid (nmol/hr/mg) (*n *= 5) in C4-2B and MR49F cells. **F** Rate of palmitate oxidation (*n *= 5), and **G** incorporation into lipid (nmol/hr/mg) (*n *= 5) in C4-2B and MR49F cells. **H** Rate of lactate oxidation (nmol/hr/mg) in C4-2B and MR49F cells (*n *= 6). **I** Rate of glutamine oxidation (*n *= 8), **J** incorporation into aqueous soluble metabolites (ASM, nmol/hr/mg) (*n *= 8) and **K** incorporation into lipid (nmol/hr/mg) (*n *= 8) in C4-2B and MR49F cells. **L** Rate of glutamine incorporation into lipid classes (nmol/hr/mg) (*n *= 8) in C4-2B and MR49F cells. **M** Ratio of oxidative and reductive utilisation of glutamine in C4-2B and MR49F (*n *= 6) as calculated by dividing *m* + *4* (oxidative) to *m* + *5* (reductive) for citrate assessed by ^13^C_5_-glutamine stable isotope tracing (24 h). TG triglyceride, DG diglyceride, PL phospholipid. Statistical significance was determined by unpaired Welch’s *t* test, as described in the Methods. *p value < 0.05. Data are presented as mean ± SEM.

In this study, we compared three LNCaP-derived prostate cancer cell lines: C4-2B cells, V16D cells, and MR49F cells (Supplementary Fig. 1A). Sensitivity to ENZ was measured with assessments of cell proliferation with increasing ENZ doses and transcript analysis of canonical AR target genes *KLK3* and *NKX3.1*. C4-2B and V16D cells were sensitive to ENZ treatment, as demonstrated by reduced cell proliferation and AR target gene expression in ENZ-treated cells (Fig. 1A, Supplementary Fig. 1B, C, E, F). MR49F cells were resistant to ENZ treatment (Fig. 1A, Supplementary Fig. 1D–F). To characterise nutrient uptake and utilisation profiles of ENZ-sensitive and ENZ-resistant prostate cancer cells, we performed ^14^C radiolabelled metabolic tracing in C4-2B and MR49F cells using four substrates (glucose, glutamine, palmitate, and lactate), measuring substrate uptake, oxidation and partitioning into various lipids (Fig. 1B). Prostate cancer cells consume nutrients from the tumour microenvironment to fuel their survival and growth [12]. Nutrient uptake rates were estimated from the combined recovery of radiolabel in the CO₂ oxidation, aqueous soluble metabolite and lipid-incorporation fractions. Glutamine was the dominant nutrient taken up by prostate cancer cells (Fig. 1C) and served as the primary oxidative substrate (Supplementary Fig. 2A), whereas palmitate contributed the most to the lipid pool (Supplementary Fig. 2B). Despite a significant increase in glucose uptake in MR49F cells compared with C4-2B cells (Fig. 1C), there were no significant increases in glucose oxidation or glucose-derived lipid incorporation (notionally de novo lipogenesis and subsequent incorporation into complex lipids) (Fig. 1D, E, Supplementary Fig. 2C). Fatty acid utilisation was similarly unchanged between the cell lines, with no differences in palmitate uptake, oxidation, or lipid storage (Fig. 1C, F, G, Supplementary Fig. 2D). There was an increase in lactate oxidation in ENZ-resistant cells compared with ENZ-sensitive cells (Fig. 1H), indicating a potential increase in reverse Warburg metabolism. ENZ resistance was associated with reprogramming of glutamine metabolism. While there was no difference in glutamine uptake between cell lines (Fig. 1C), glutamine oxidation was increased by ~10% (Fig. 1I), glutamine incorporation into aqueous soluble metabolites was reduced by ~13% (Fig. 1J), and incorporation into lipids was reduced by ~70% in ENZ-resistant vs ENZ-sensitive cells (Fig. 1K, L). This shift was not indicative of a global suppression of de novo lipogenesis, as glucose-derived lipid synthesis was maintained in MR49F cells (Fig. 1E), supporting the likelihood of selective attenuation of glutamine-fuelled lipogenesis. Indeed, further tracing using ^13^C-glutamine showed an increase in the rate of glutamine metabolised through the oxidative TCA cycle at the expense of the reductive carboxylation pathway in MR49F cells (Fig. 1M, Supplementary Fig. 2E).

Although these radiotracer studies revealed measurable changes in nutrient handling between ENZ-sensitive and ENZ-resistant cells, overall differences were relatively subtle. The changes observed in glucose, glutamine and lactate utilisation nonetheless demonstrate metabolic reprogramming in ENZ-resistant cells. While ^14^C metabolic tracing provides pathway-specific information and rates of substrate metabolism, we also sought to resolve broader changes across interconnected pathways, metabolite pools, and regulatory nodes of metabolism.

### Multi-omic characterisation of ENZ-sensitive and ENZ-resistant prostate cancer cells identifies metabolic reprogramming as a hallmark of therapy resistance

**Fig. 2:**
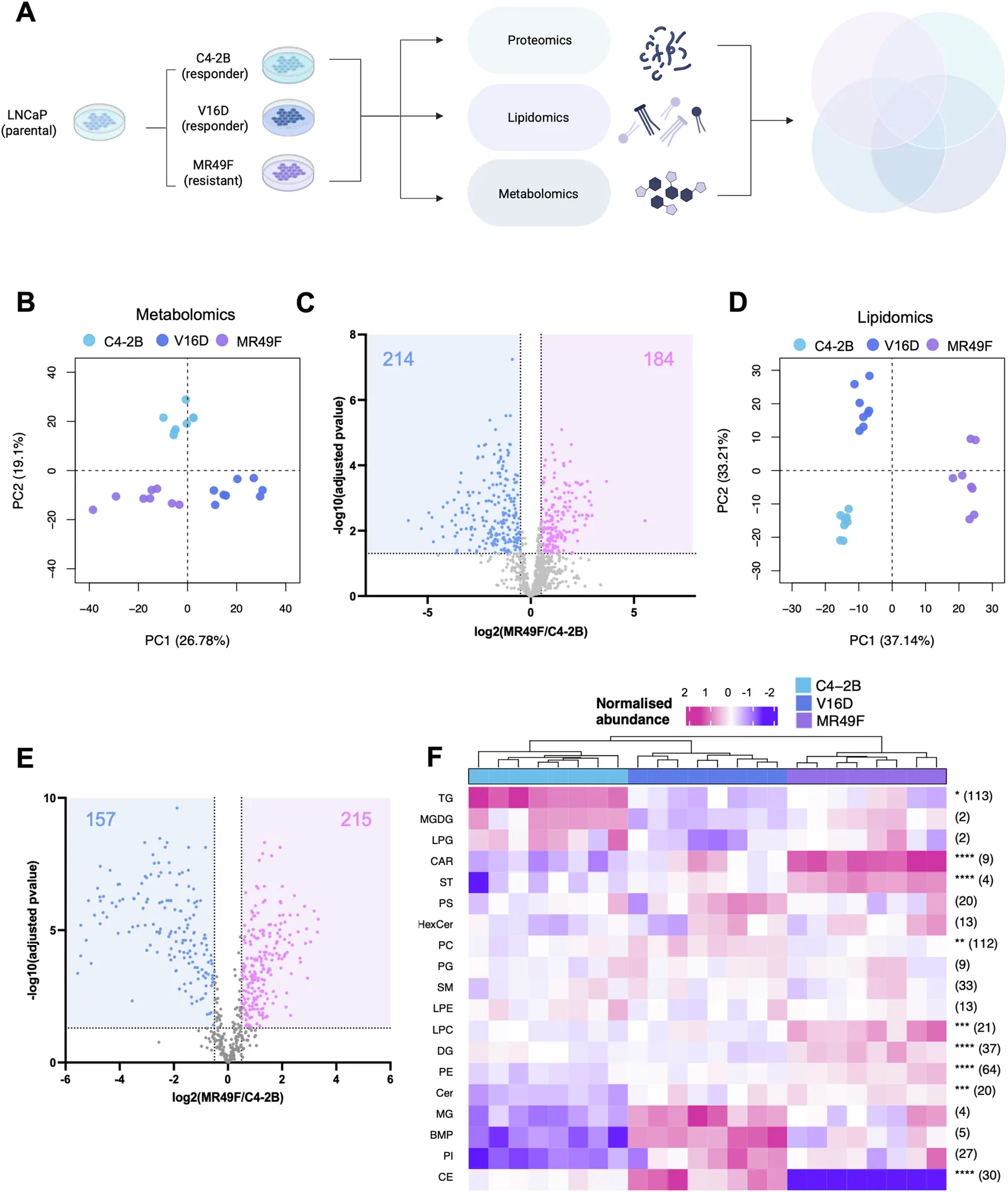
Lipidomic and metabolomic profiling of ENZ-sensitive and ENZ-resistant cells shows metabolic programming is a hallmark of ENZ resistance in prostate cancer cells. **A** Schematic showing experimental approach that involves multi-omic profiling of C4-2B, V16D and MR49F cells using proteomics, lipidomics, and metabolomics. Created in BioRender.com. **B** Principal component analysis (PCA) of C4-2B (blue, *n *= 8), V16D (dark blue, *n *= 8) and MR49F (purple, *n *= 8) metabolomic data with explained variance (%) shown for PC1 and PC2. **C** Volcano plot of log_2_(fold change) expression of metabolites against -log_10_(adjusted *p* value) comparing MR49F cells and C4-2B cells. **D** Principal component analysis (PCA) of C4-2B (blue, *n *= 8), V16D (dark blue, *n *= 8) and MR49F (purple, *n *= 8) lipidomic data with explained variance (%) shown for PC1 and PC2. **E** Volcano plot of log_2_(fold change) expression of lipids against -log_10_(adjusted *p* value) comparing MR49F cells and C4-2B cells. **F** Heatmap of lipid types (median peak area) in C4-2B, V16D and MR49F cells assessed by lipidomic analysis. Individual samples and lipid types were arranged by unsupervised clustering. TG triglyceride, DG diglyceride, PE phosphatidylethanolamine, PI phosphatidylinositol, PC phosphatidylcholine, PS phosphatidylserine, PG phosphatidylglycerol, LPC lysophosphatidylcholine, LPG lysophosphatidylglycerol, LPE lysophosphatidylethanolamine, Cer ceramide, CAR acyl-carnitine, CE cholesterol ester, ST sterol, SM sphingomyelin, HexCer hexosylceramide, MG monoglyceride, MGDG monogalactosyldiacylglycerol, BMP bis(monoacylglycero)phosphate. Statistical significance was determined by unpaired two-tailed Welch’s *t* test comparing MR49F with both C4-2B and V16D cells with multiple testing correction, as described in the Methods.

To get a more comprehensive understanding of the metabolic changes occurring with ENZ resistance, we performed metabolomic, lipidomic, and proteomic analyses comparing C4-2B, an additional ENZ-sensitive cell line V16D (Fig. 1A), and MR49F cells (Fig. 2A, Supplementary Table 1).

Targeted metabolomic analysis revealed marked differences in the abundance of polar metabolites between C4-2B, V16D and MR49F cells, as shown by principal component analysis (PCA) (Fig. 2B). Of the 1162 metabolites that were detected (Supplementary Table 1), 214 (18%) were increased and 184 (15%) were reduced in ENZ-resistant cells relative to C4-2B cells (Fig. 2C).

Untargeted lipidomics analysis detected 578 lipid species in all three cell lines (Supplementary Table 1) and marked differences in the cellular lipidomes of ENZ-sensitive and ENZ-resistant cells (Fig. 2D). Two hundred and fifteen (37%) lipid species were increased in MR49F cells compared to C4-2B cells, while 157 lipids were decreased (27%) (Fig. 2E). Assessing the abundance of lipid classes, ENZ-resistant cells had lower levels of cholesterol esters compared with ENZ-sensitive cells (Fig. 2F). Several lipid classes were increased in ENZ-resistant MR49F cells, including sterol lipids (ST), acylcarnitines (CAR), diglycerides (DG), phosphatidylethanolamines (PE), and lysophosphatidylcholines (LPC) (Fig. 2F).

**Fig. 3:**
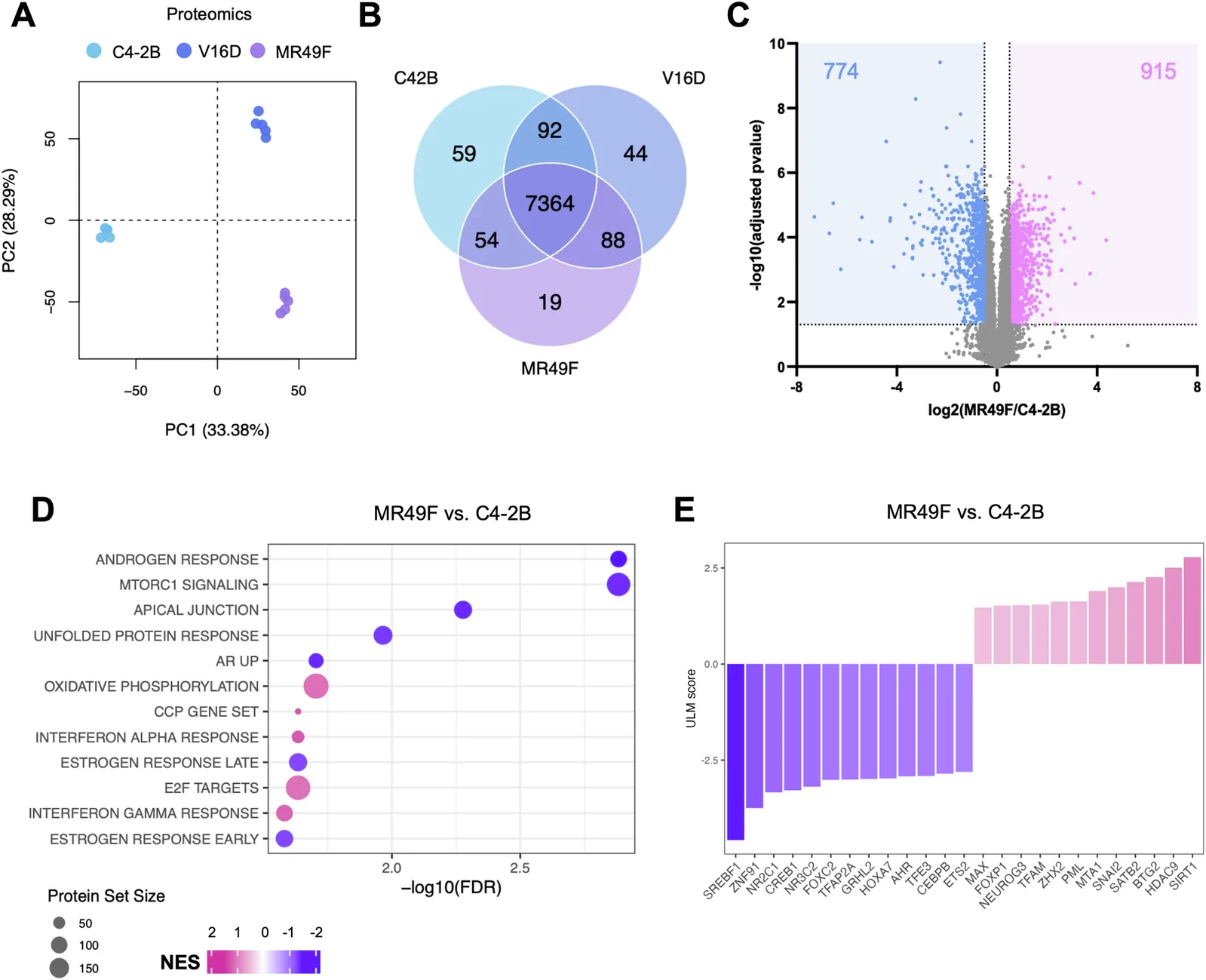
Proteomic profiling of ENZ-sensitive and ENZ-resistant cells shows reprogramming of the protein landscape in ENZ resistance in prostate cancer cells. **A** Principal component analysis (PCA) of C4-2B (blue, *n *= 5), V16D (dark blue, *n *= 5) and MR49F (purple, *n *= 5) proteomic data with explained variance (%) shown for PC1 and PC2. **B** Venn diagram of proteins detected by mass spectrometry in C4-2B, V16D and MR49F cells. **C** Volcano plot of log_2_(fold change) of proteins against -log_10_(adjusted *p* value) comparing MR49F cells and C4-2B cells. **D** Protein set enrichment analysis (PSEA) of C4-2B and MR49F cells using HALLMARK, KEGG, and selected curated gene sets. **E** Transcription factor (TF) activity was inferred from proteomic differential expression data using the ULM method. Bars represent inferred TF activity scores, with colour indicating direction and magnitude of activity. The top 25 ranked TFs (*p* value < 0.05) are shown. Statistical significance was determined by unpaired two-tailed Welch’s *t* test with multiple testing correction, as described in the Methods.

To elucidate the mechanisms driving metabolic reprogramming in ENZ resistance, we complemented our lipidomic and metabolomic analyses with assessment of the cellular proteome. PCA showed distinct clustering of all three cell lines (Fig. 3A), demonstrating that wide proteomic reprogramming occurs with ENZ resistance and between ENZ-sensitive models. Untargeted proteomic analysis identified 7364 proteins in all cell lines (Supplementary Table 1), as well as 92 proteins that were exclusively detected in ENZ-sensitive cell lines, and 19 proteins that were exclusively detected in MR49F cells (Fig. 3B). We found that 915 (12%) proteins were upregulated, and 774 (10%) proteins were downregulated in MR49F cells when compared to C4-2B cells (Fig. 3C). Parallel differential analyses comparing MR49F with V16D similarly demonstrated extensive remodelling across the metabolomic, lipidomic and proteomic datasets, providing orthogonal support for metabolic and proteomic alterations associated with the ENZ-resistant phenotype (Supplementary Fig. 3A–E).

Protein set enrichment analysis (PSEA) identified down-regulation of proteins associated with hormone signalling in ENZ-resistant prostate cancer cells, including “androgen response”, “estrogen response early” and “estrogen response late” (Fig. 3D). “mTORC1 signalling” was also enriched in the proteins down-regulated in MR49F cells relative to C4-2B cells (Fig. 3D). Alternatively, inflammatory pathways including “interferon alpha response” and “interferon gamma response” were enriched (Fig. 3D). The “oxidative metabolism” pathway was enriched in the proteins up-regulated in MR49F cells, suggesting that ENZ resistance is associated with changes in oxidative metabolism (Fig. 3D). Transcription factor (TF) activity profiling of the proteomic dataset comparing MR49F and C4-2B cells revealed suppression of target genes downstream of sterol regulatory element-binding transcription factor 1 (SREBF1), a master lipid metabolism regulator, which showed the lowest inferred activity score (Fig. 3E).

Overall, these multi-omic assessments showcase consistent metabolic and proteomic reprogramming in ENZ-resistant prostate cancer. To systematically identify metabolic pathways associated with actionable vulnerabilities, we prioritised alterations that distinguished MR49F cells from both ENZ-sensitive models, C4-2B and V16D, rather than changes observed in only a single pairwise comparison. We then focused on pathways that showed convergent dysregulation across multiple omic datasets, for example, concordant changes in regulatory proteins together with shifts in lipids or polar metabolites in biological pathways. This cross-platform concordance increased confidence that observed alterations in individual modalities reflected true biological reprogramming. Finally, candidate pathways were evaluated based on whether their biochemical function has been shown to be essential for tumour cell survival or proliferation, such that perturbing the pathway limits metabolic flexibility or imposes cytotoxic stress. We therefore pursued two parallel functional investigations arising from the multi-omic analyses: the consequences of altered PUFA metabolism identified through lipidomics, and the significance of altered ammonium handling identified through polar metabolomics.

### ENZ-resistant prostate cancer cells have reduced levels of polyunsaturated fatty acid (PUFA) synthesis

**Fig. 4:**
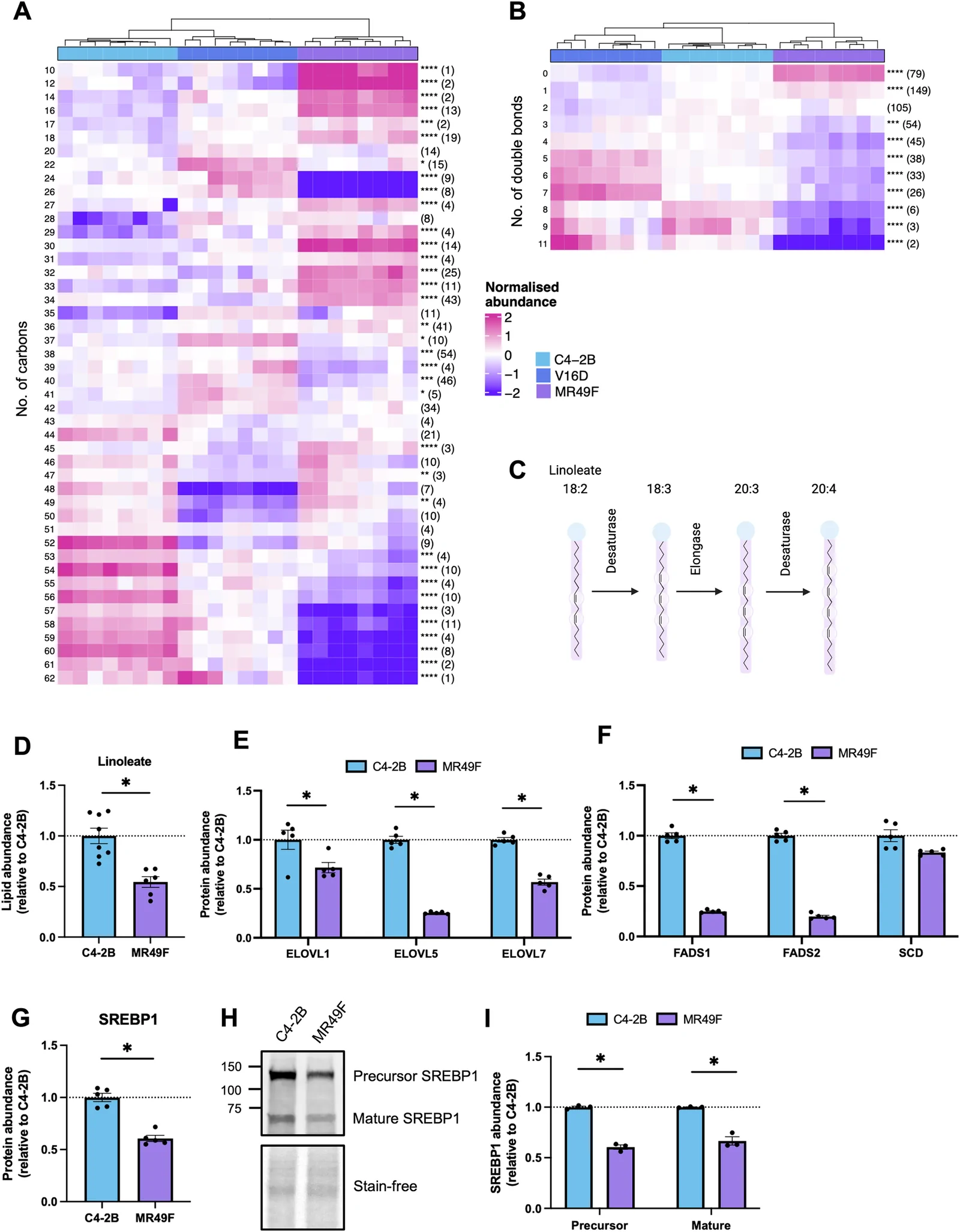
Reduced polyunsaturated fatty acid (PUFA) abundance in ENZ-resistant prostate cancer cells. **A** Heatmap of abundance of all detected lipids according to the number of carbons present in fatty acid chains in C4-2B (*n *= 8), V16D (*n *= 8) and MR49F (*n *= 8) cells. Individual samples were arranged by unsupervised clustering. **B** Heatmap of abundance of all detected lipids according to the number of double bonds present in fatty acid chains in C4-2B (*n *= 8), V16D (*n *= 8) and MR49F (*n *= 8) cells. Individual samples were arranged by unsupervised clustering. **C** Schematic illustrating synthesis of polyunsaturated fatty acids (PUFAs). Created in BioRender.com. **D** Linoleate (FA 18:2) abundance in C4-2B and MR49F cells (*n *= 8) assessed by lipidomic analysis. **E** Protein content of elongase enzymes ELOVL1, ELOVL5 and ELOVL7 in C4-2B and MR49F cells (*n *= 5). **F** Protein content of desaturase enzymes SCD, FADS1 and FADS2 in C4-2B and MR49F cells (*n *= 5). **G** Protein content of SREBP1 in C4-2B and MR49F cells (*n *= 5) assessed by proteomics analysis. **H** Representative immunoblot (cropped) showing SREBP1 protein in C4-2B and MR49F cells (*n *= 3). **I** Quantification of precursor and mature SREBP1 protein in C4-2B and MR49F cells from immunoblot in (**H**). Statistical significance was determined by unpaired two-tailed Welch’s *t* test with or without multiple testing correction, as described in the Methods. *p value < 0.05. Data are presented as mean ± SEM.

Lipids differ in their structure, depending on the length of the fatty acyl chain and the number of double bonds present. Analysis of the lipidomic dataset revealed a striking shift in the abundance of lipid species according to the length and desaturation of their fatty acids. Generally, there was enrichment in lipid species with shorter fatty acyl chains and a striking depletion in lipid species with long fatty acyl chains in MR49F cells compared with C4-2B and V16D cells (Fig. 4A).

The number of double bonds in a lipid represents its saturation, with saturated fatty acids (SFA) containing no double bonds, monounsaturated fatty acids (MUFAs) containing one double bond, and polyunsaturated fatty acids (PUFAs) containing two or more double bonds. When compared with C4-2B cells and V16D cells, there was a substantial depletion of lipids with three or more double bonds in MR49F cells (Fig. 4B), which is highly indicative of reduced PUFA abundance. This was matched with a concomitant enrichment in SFAs and MUFAs (Fig. 4B, C). This pattern was specific to certain lipid classes, including cholesterol esters, phosphatidylcholines, and triglycerides (Supplementary Fig. 4A).

PUFAs are generated through the elongation and desaturation of the precursor fatty acid linoleic acid or ${\rm{\alpha }}$-linolenic acid which are taken up from the extracellular environment [36] (Fig. 4C). We found that linoleic acid levels were reduced in MR49F cells compared with C4-2B cells (Fig. 4D), but not relative to V16D cells (Supplementary Fig. 4B). ${\rm{\alpha }}$-linolenic acid was not detected in our study. The synthesis of longer chain PUFAs from linoleate requires the alternating action of two enzyme families: elongases (ELOVLs) and desaturases [36] (Fig. 4C). All ELOVL enzymes detected in our proteomics analysis, ELOVL1, ELOVL5 and ELOVL7, were decreased in MR49F cells compared with C4-2B cells (Fig. 4E). Yet, when comparing MR49F cells with V16D cells, only ELOVL5 was reduced (Supplementary Fig. 4C). Other ELOVL isoforms, ELOVL2, ELOVL3, ELOVL4 and ELOVL6, were not detected in the proteomics analysis. Likewise, the lipid desaturase enzymes FADS1 and FADS2 were markedly decreased in MR49F cells compared with both C4-2B cells and V16D cells (Fig. 4F, Supplementary Fig. 4D). Stearoyl-CoA desaturase 1 (SCD1), which catalyses the conversion of MUFAs from SFAs, tended (*P* = 0.07) to be reduced in MR49F cells relative to C4-2B cells, but not V16D cells (Fig. 4F, Supplementary Fig. 4D), while SCD5 was exclusively expressed in MR49F cells (data not shown).

Many elongase and desaturase enzymes are transcriptionally regulated by sterol regulatory element-binding transcription factor 1 (SREBP1), encoded by *SREBF1*, which was the transcription factor with the lowest activity score in the proteomic dataset (Fig. 3E) [37]. Both the mature and precursor forms of SREBP1 were reduced in MR49F cells compared with C4-2B cells (Fig. 4G–I). Overall, these findings suggest that ENZ-resistant cells down-regulate PUFA synthesis, likely via a reduction in SREBP1-mediated transcriptional initiation of elongase and desaturase enzymes. We next sought to investigate the functional consequences of reduced PUFA abundance in ENZ-resistant cells. For these experiments, we used C4-2B cells as the ENZ-sensitive model, owing to their greater sensitivity to ENZ treatment than V16D cells, and MR49F cells as the ENZ-resistant model.

### ENZ-resistant cells are resistant to lipid peroxidation and ferroptosis

**Fig. 5:**
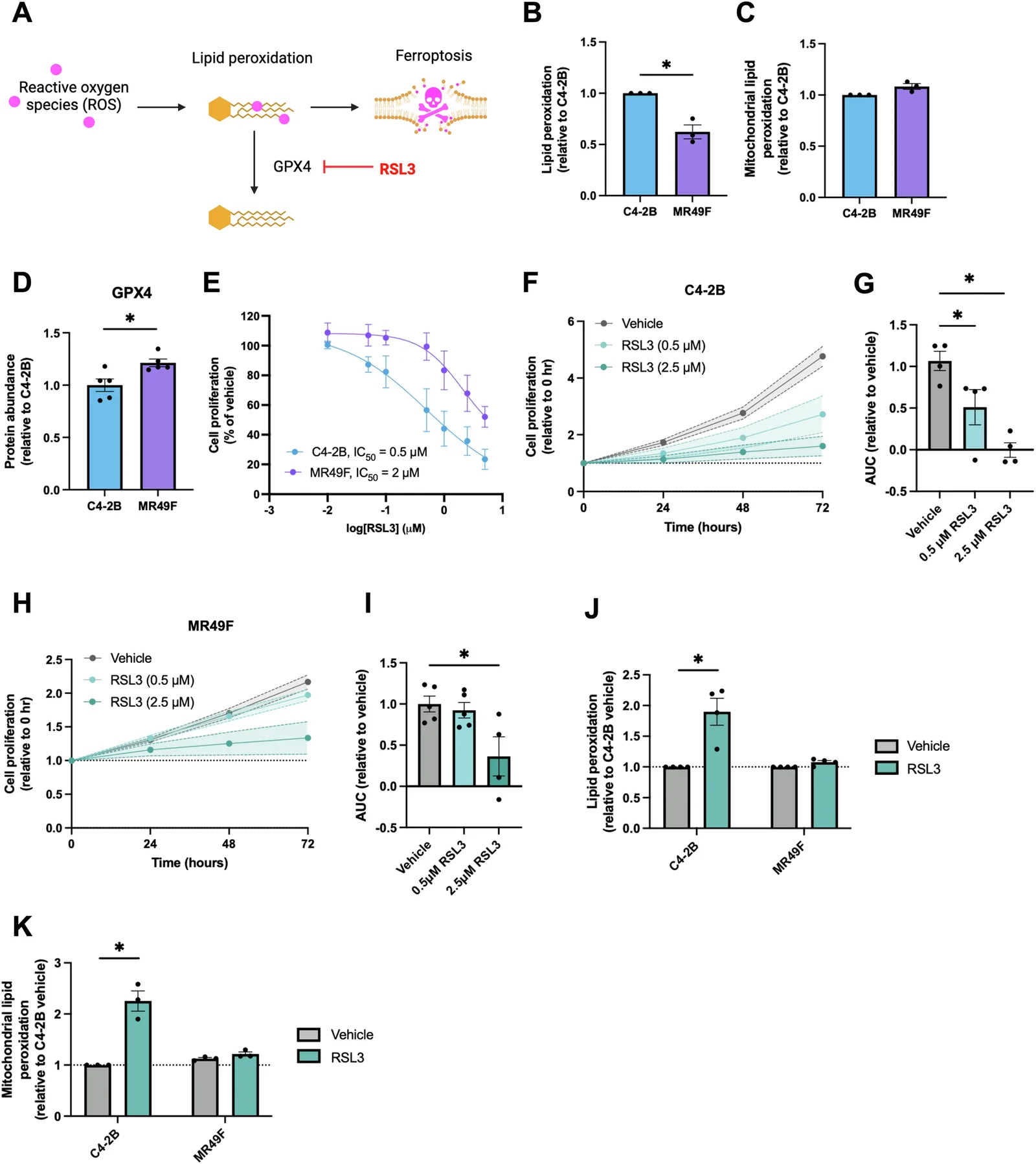
ENZ-resistant prostate cancer cells are more resistant to oxidative stress and lipid peroxidation. **A** Schematic illustrating process of lipid peroxidation and detoxification of ROS and lipid peroxides. Created in BioRender.com. **B** Lipid peroxidation levels in C4-2B and MR49F cells assessed by flow cytometry (C11-BODIPY, 2.5 ${\rm{\mu }}$M) (*n *= 3). **C** Mitochondrial lipid peroxidation levels in C4-2B and MR49F cells assessed by flow cytometry (MitoPerOx, 2.5 ${\rm{\mu }}$M) (*n *= 3). **D** Protein content of GPX4 in C4-2B and MR49F cells (*n *= 5). **E** Dose-response curve analysis showing cell proliferation of C4-2B and MR49F cells treated with increasing doses of RSL3 for 72 h. **F** Growth curves of C4-2B cells treating with vehicle or RSL3 (0.5 ${\rm{\mu }}$M and 2.5 ${\rm{\mu }}$M) over 72 hours, measured by IncuCyte SX5 live-cell imaging (*n *= 4). **G** Area under curve (AUC, incremental) analysis of growth curves in (**F**). **H** Growth curves of MR49F cells treating with vehicle or RSL3 (0.5 ${\rm{\mu }}$M and 2.5 ${\rm{\mu }}$M) over 72 h, measured by IncuCyte SX5 live-cell imaging (*n *= 5). **I** Area under curve (AUC, incremental) analysis of growth curves in (**H**). **J** Lipid peroxidation levels in C4-2B and MR49F cells treated with vehicle or RSL3 (2.5 ${\rm{\mu }}$M) for 2 h assessed by flow cytometry (C11-BODIPY, 2.5 ${\rm{\mu }}$M) (*n *= 4). **K** Mitochondrial lipid peroxidation levels in C4-2B and MR49F treated with vehicle or RSL3 (2.5 ${\rm{\mu }}$M) for 2 h assessed by flow cytometry (MitoPerOx, 2.5 ${\rm{\mu }}$M) (*n *= 3). Statistical significance was determined using an unpaired Welch’s *t* test, as described in the Methods. *p value < 0.05. Data are presented as mean ± SEM.

PUFAs are highly susceptible to damaging insults by reactive oxygen species (ROS). This process, known as lipid peroxidation, disrupts cell membrane integrity and causes a form of cell death called ferroptosis (Fig. 5A) [38]. Based on the findings above, we hypothesised that ENZ-resistant prostate cancer cells suppress PUFA synthesis to protect against oxidative stress and ferroptosis. Notably, while ENZ exerts its anti-tumour effects primarily through AR blockade, emerging evidence suggests that this is accompanied by ROS accumulation and lipid peroxidation, indicating that oxidative stress can contribute to treatment response in some contexts [28, 39]. Lipid peroxidation was significantly reduced in MR49F cells compared with C4-2B cells (Fig. 5B). To test whether this difference reflected mitochondrial oxidative damage, a major source of ROS and a key site of ferroptosis-associated lipid oxidation [40], we next quantified mitochondrial lipid peroxidation. Mitochondrial lipid peroxidation levels were comparable between the two cell lines (Fig. 5C).

Cells primarily detoxify lipid peroxides through the action of the antioxidant enzyme glutathione peroxidase 4 (GPX4) [41] (Fig. 5A). GPX4 was increased in MR49F cells relative to C4-2B cells (Fig. 5D). Thus, we utilised RSL3, a highly specific GPX4 inhibitor, to measure the sensitivity of C4-2B and MR49F cells to ferroptosis. Dose-response curve and growth curve analyses revealed that C4-2B cells were more sensitive to the anti-proliferative effects of RSL3 treatment compared with MR49F cells (Fig. 5E–I). The same was observed when treating cells with the GPX4-independent oxidative stress inducer, the system x_c_ transporter (SLC7A11) inhibitor erastin (Supplementary Fig. 5A–F). Acute treatment with RSL3 increased peroxidation levels in C4-2B, but not MR49F cells (Fig. 5J, Supplementary Fig. 5G). Notably, RSL3 treatment resulted in an increase in mitochondria-localised lipid peroxidation in C4-2B cells, but not in MR49F cells (Fig. 5K, Supplementary Fig. 5H). Therefore, ENZ-resistant cells have lower lipid peroxidation levels and are more resistant to GPX4-dependent induction of lipid peroxidation and ferroptosis compared with ENZ-sensitive cells.

Previous studies have reported that ENZ treatment itself can increase ROS levels and lipid peroxidation, leading to the proposal that ENZ may suppress prostate cancer cell proliferation [28, 33], at least in part, through the induction of ferroptosis. To determine whether this response occurred in our experimental system, C4-2B cells were treated with ENZ for two or five days before lipid peroxidation was quantified. ENZ treatment did not significantly alter lipid peroxidation at either time point (Supplementary Fig. 5I).

### PUFA supplementation re-sensitises ENZ-resistant prostate cancer cells to lipid peroxidation and ferroptosis

**Fig. 6:**
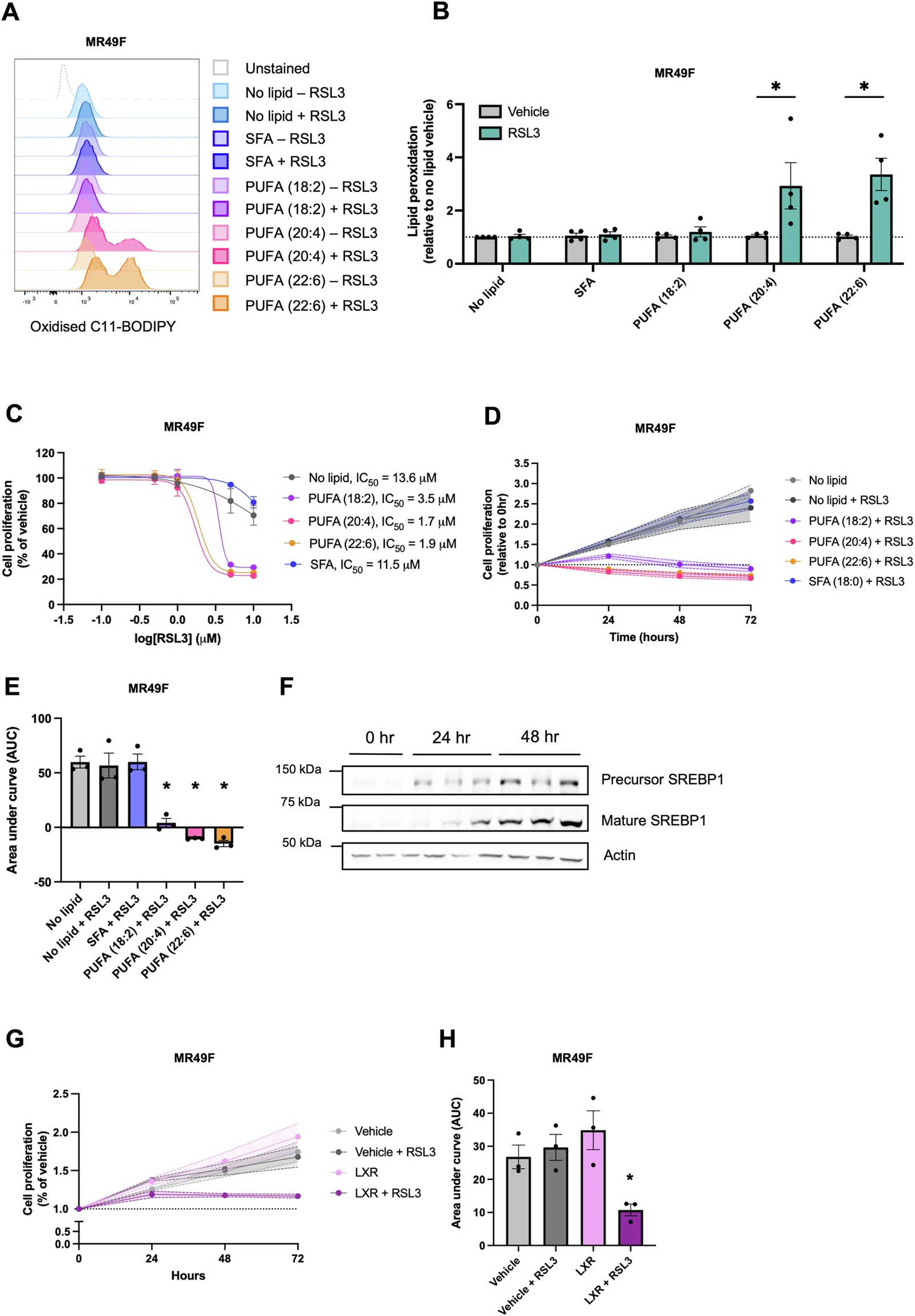
PUFA supplementation sensitises ENZ-resistant prostate cancer cells to lipid peroxidation and ferroptosis. **A** Representative histograms of C11-BODIPY (B525) intensity in MR49F cells pre-treated with no lipid (BSA only), palmitate (SFA, 10 ${\rm{\mu }}$M, 16 h), linoleate (PUFA (18:2), 10 ${\rm{\mu }}$M, 16 h), arachidonic acid (PUFA (20:4), 10 ${\rm{\mu }}$M, 16 h), or docosahexaenoic acid (PUFA (22:6), 10 ${\rm{\mu }}$M, 16 h) and treated with vehicle or RSL3 (2.5 ${\rm{\mu }}$M, 2 h). **B** Lipid peroxidation levels in MR49F cells pre-treated with no lipid (BSA only), palmitate (SFA, 10 ${\rm{\mu }}$M, 16 h), linoleate (PUFA (18:2), 10 ${\rm{\mu }}$M, 16 h), arachidonic acid (PUFA (20:4), 10 ${\rm{\mu }}$M, 16 h), or docosahexaenoic acid (PUFA (22:6), 10 ${\rm{\mu }}$M, 16 h), and treated with vehicle or RSL3 (2.5 ${\rm{\mu }}$M, 2 h) (*n *= 4). **C** Dose-response curve analysis showing cell proliferation of MR49F cells pre-treated with no lipid (BSA only), palmitate (SFA, 10 ${\rm{\mu }}$M, 16 h), linoleate (PUFA (18:2), 10 ${\rm{\mu }}$M, 16 h), arachidonic acid (PUFA (20:4), 10 ${\rm{\mu }}$M, 16 h), or docosahexaenoic acid (PUFA (22:6), 10 ${\rm{\mu }}$M, 16 h), and treated with vehicle or RSL3 (0.1, 0.5, 1, 5 and 10 ${\rm{\mu }}$M) following 72 hours (*n *= 3). **D** Growth curves of MR49F cells pre-treated with no lipid (BSA only), palmitate (SFA, 10 ${\rm{\mu }}$M, 16 h), linoleate (PUFA (18:2), 10 ${\rm{\mu }}$M, 16 h), arachidonic acid (PUFA (20:4), 10 ${\rm{\mu }}$M, 16 h), or docosahexaenoic acid (PUFA (22:6), 10 ${\rm{\mu }}$M, 16 h), and treated with vehicle or RSL3 (5 ${\rm{\mu }}$M) over 72 h, measured by IncuCyte SX5 live-cell imaging (*n *= 3). **E** Area under curve (AUC, incremental) analysis of growth curves in (**D**). **F** Immunoblot (cropped) of precursor and mature SREBP1 in MR49F cells treating with 0.5 ${\rm{\mu }}$M GW3965 0, 24 and 48 h. **G** Growth curves of MR49F cells pre-treated with vehicle or LXR agonist GW3965 (0.5 ${\rm{\mu }}$M, 16 h) and treated with vehicle or RSL3 (5 ${\rm{\mu }}$M) over 72 h, measured by IncuCyte SX5 live-cell imaging (*n *= 3). **H** Area under curve (AUC, incremental) analysis of growth curves in (**F**). Statistical significance was determined using an unpaired Welch’s *t* test, as described in the Methods. *p value < 0.05. Data are presented as mean ± SEM.

To assess whether the reduction in PUFA abundance was responsible for the increased resistance to lipid peroxidation and ferroptosis in ENZ-resistant cells, we supplemented MR49F cells with three PUFAs that differ in length and desaturation: linoleate (PUFA 18:2), arachidonic acid (AA, PUFA 20:4), and docosahexaenoic acid (DHA, PUFA 22:6). We also included a saturated fatty acid, palmitate (SFA, 16:0) that does not contain double bonds. Following 16 h of exposure to the individual fatty acids, cells were treated with vehicle or RSL3 and lipid peroxidation was assessed using flow cytometry. As shown in earlier experiments (Fig. 5J), treating MR49F with RSL3 did not affect lipid peroxidation (Fig. 6A, B). There was no difference in lipid peroxidation levels with RSL3 in MR49F cells that had been exposed to palmitate or linoleate (Fig. 6A, B). In contrast, MR49F cells exposed to AA and DHA were sensitised to RSL3-induced lipid peroxidation, increasing by 2.9- and 3.5-fold, respectively (Fig. 6A, B). This suggests that highly unsaturated PUFAs containing four or more double bonds re-sensitise ENZ-resistant cells to RSL3-induced lipid peroxidation.

Moreover, in MR49F cells pre-treated with DHA and AA, RSL3-induced lipid peroxidation was associated with an increase in the viability stain DAPI (Supplementary Fig. 6F, H), suggesting that PUFA supplementation increases both RSL3-induced lipid peroxidation and cell death, likely attributed to ferroptosis.

Cell proliferation studies assessed whether PUFA supplementation re-sensitises ENZ-resistant prostate cancer cells to ferroptosis. Overall, dose-response curve analysis revealed that supplementing MR49F cells with all three PUFAs (linoleate, AA, and DHA) increased sensitivity of the cells to RSL3-induced growth inhibition (Fig. 6C–E). RSL3 treatment of MR49F cells that had been pre-treated with PUFAs led to a ~70% reduction in proliferation (Fig. 6C). This was specific to PUFA supplementation, as treatment of MR49F cells with the saturated fatty acid palmitate did not affect RSL3 sensitivity (Fig. 6C). The same findings were observed when performing growth curve analysis (Fig. 6D, E).

To ascertain whether the growth-restricting effects of PUFA supplementation were specific to ENZ-resistant cells, we repeated these experiments in C4-2B cells. Growth curve analysis showed that supplementation of C4-2B cells with PUFAs did not result in changes in RSL3-induced growth inhibition (Supplementary Fig. 7A, B), likely because C4-2B cells, even in the absence of exogenous fatty acids, were highly sensitive to RSL3-induced ferroptosis. Dose-response curve analysis showed that supplementation of C4-2B cells with certain PUFAs increased sensitivity of the cells to lower doses of RSL3, which was not observed with SFA supplementation (Supplementary Fig. 7C). PUFA supplementation failed to restore erastin sensitivity in C4-2B or MR49F cells (Supplementary Fig. 7D, E), indicating that PUFA effects on oxidative stress are GPX4-dependent.

These data demonstrate that uptake of exogenous PUFAs is sufficient to re-sensitise ENZ-resistant cells to ferroptosis. In addition to reduced PUFA abundance, we also observed lower levels of de novo PUFA synthesis enzymes and SREBP1 (Fig. 4F–I). Therefore, we tested whether restoring SREBP1 levels, and thus de novo PUFA synthesis, was able to re-sensitise ENZ-resistant cells to RSL3. To achieve this, we treated MR49F cells with the liver X receptor (LXR) agonist GW3965 to increase levels of mature SREBP1, elongase/desaturase expression and PC PUFA abundance (Fig. 6F, Supplementary Fig. 8). Treating MR49F cells with the LXR agonist increased the sensitivity of the cells to RSL3 treatment, with the combination substantially blocking cell proliferation (Fig. 6G, H). Together, this indicates that the regulation of PUFA synthesis by SREBP1 plays an important role in protecting ENZ-resistant prostate cancer cells from ferroptosis.

### ENZ-resistant prostate cancer cells have a higher capacity to de-toxify ammonium

**Fig. 7:**
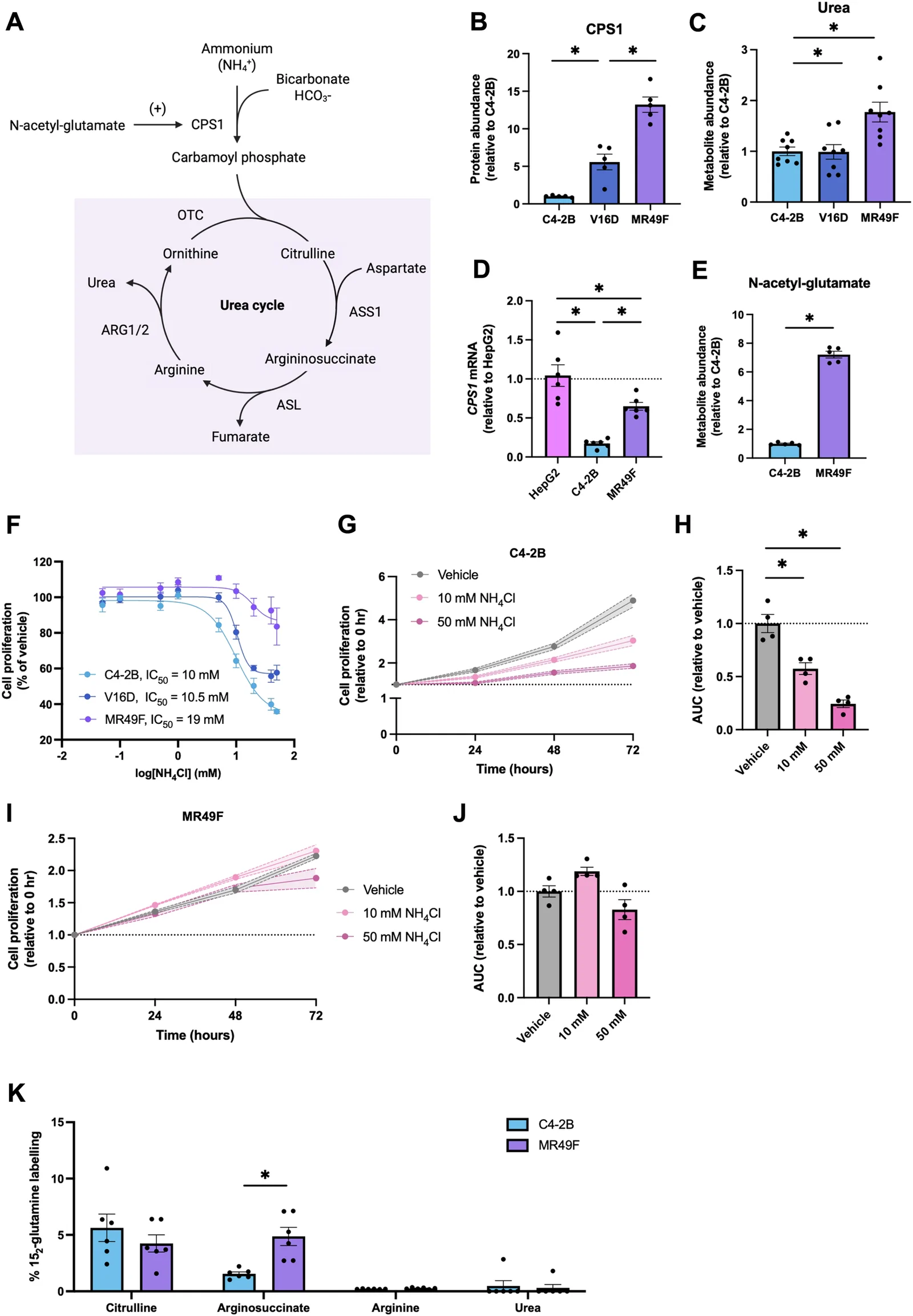
ENZ-resistant prostate cancer cells have a higher capacity to de-toxify ammonium through carbamoyl phosphate synthetase 1 (CPS1). **A** Schematic illustrating the detoxification of ammonium to carbamoyl phosphate by CPS1, requiring N-acetyl-glutamate as an allosteric activator. Carbamoyl phosphate is then metabolised through the urea cycle to produce urea. Created in BioRender.com. **B** Protein content of carbamoyl phosphate synthetase 1 (CPS1) in C4-2B, V16D and MR49F cells (*n *= 5). **C** Urea abundance in C4-2B, V16D and MR49F cells (*n *= 8). **D** mRNA expression of *CPS1* in HepG2, C4-2B and MR49F cells (*n *= 6). **E** N-acetyl-glutamate (NAG) abundance in C4-2B and MR49F cells assessed by targeted metabolomics (*n *= 5). **F** Dose-response curve (non-linear fit) showing percentage cell proliferation of C4-2B (blue), V16D (dark blue) and MR49F (purple) cells at increasing concentrations of NH_4_Cl (0.05, 0.1, 0.5, 1, 5, 10, 20, 40, 50 mM) following 72 hours (*n *= 4). **G** Cell proliferation of C4-2B cells treated with vehicle, 10 mM NH_4_Cl or 50 mM NH_4_Cl over 72 hours (*n *= 4). **H** Area under curve (AUC, incremental) analysis of growth curves in (**G**). **I** Cell proliferation of MR49F cells treated with vehicle, 10 mM NH_4_Cl or 50 mM NH_4_Cl over 72 h (*n *= 5). **J** Area under curve (AUC, incremental) analysis of growth curves in (**I**). **K** Percentage labelling (%) of urea cycle intermediates citrulline, arginosuccinate, arginine and urea with ^13^C_5_^15^N_2_-glutamine following 24 h in C4-2B and MR49F cells (*n *= 6). Statistical significance was determined using a one-way ANOVA or unpaired Welch’s *t* test, as described in the Methods. *p value < 0.05. Data are presented as mean ± SEM.

In parallel with our functional investigation of the lipidomic phenotype, we next examined whether alterations identified in the polar metabolomic dataset revealed additional metabolic adaptations in ENZ-resistant cells, focusing on the marked changes associated with ammonium detoxification. Carbamoyl phosphate synthetase 1 (CPS1) is the first and rate-limiting enzyme involved in the detoxification of ammonium (NH_4_^+^), an inorganic derivative of nitrogen that is produced as a by-product of amino acid metabolism [42] (Fig. 7A). CPS1 converts ammonium to carbamoyl phosphate, which is metabolised through the urea cycle to generate urea that can be removed from the cell (Fig. 7A). CPS1 was increased by a striking ~14-fold in ENZ-resistant prostate cancer cells compared to C4-2B cells (Fig. 7B), and ~2.5-fold relative to V16D cells. Moreover, we detected higher levels of urea in MR49F cells (Fig. 7C). Together, this indicated altered flux through the CPS1 reaction and possible implications for enhanced ammonium detoxification in ENZ-resistant cells.

When accumulating to excess levels, ammonium imbalances cellular pH, induces oxidative stress and causes mitochondrial dysfunction, resulting in cell death [43]. Cancer cells protect themselves against supraphysiological ammonium exposure by using CPS1 to convert ammonium to a more stable form that can be removed from the cell [14]. The major organ that detoxifies systemic ammonium is the liver [14], prompting the measurement of CPS1 expression in prostate cancer cells relative to HepG2 liver cancer cells. As expected, *CPS1* transcript levels were significantly lower in C4-2B cells relative to HepG2 cells (Fig. 7D). *CPS1* mRNA in MR49F cells was ~4.5-fold greater than C4-2B cells and reached nearly 65% of the mRNA levels observed in HepG2 cells, suggesting that ENZ-resistant prostate cancer cells may maintain sufficient *CPS1* expression to support its physiological function (Fig. 7D). Additionally, CPS1 is allosterically activated by the metabolite N-acetyl-glutamate, which we detected using targeted metabolomics analysis. NAG levels were markedly elevated in MR49F cells relative to C4-2B cells (Fig. 7E). This led us to hypothesise that ENZ-resistant cells have an increased capacity to detoxify ammonium compared to C4-2B cells. To test this, C4-2B, V16D and MR49F cells were exposed to increasing concentrations of ammonium chloride (NH_4_Cl), to increase intracellular ammonium [44], and cell proliferation was assessed. Dose-response curve analysis showed that C4-2B and V16D cells were sensitive to NH_4_Cl treatment compared with MR49F cells (Fig. 7F), with cell proliferation attenuated by >85% at the highest NH_4_Cl dose in C4-2B cells (Fig. 7G, H). In contrast, MR49F cell proliferation was not significantly affected by NH_4_Cl exposure relative to vehicle treatment (Fig. 7I, J). Therefore, we speculated that ENZ-resistant prostate cancer cells have a higher capacity to detoxify ammonium, which may serve as a targetable vulnerability to induce nitrogen stress and cell death. As in our previous functional investigation, subsequent experiments were performed in C4-2B and MR49F cells as the ENZ-sensitive and ENZ-resistant models, respectively. To test whether the urea cycle downstream of CPS1 is active in prostate cancer cells, we performed stable isotope tracing of ^15^N_2_-labelled glutamine (24 h) into urea cycle intermediates: citrulline, arginosuccinate, arginine and urea. Glutamine is the major source of cellular ammonium, with previous studies reporting that up to ~55% of ammonium secreted from cultured cancer cells is derived from glutamine [45], which we showed to be a major nutrient substrate in prostate cancer cells. In both cells lines, a small proportion of urea cycle metabolites were labelled with glutamine-derived nitrogen groups. Approximately 5% of citrulline and arginosuccinate were labelled with glutamine-derived ^15^N (Fig. 7K). There was a significant increase in ^15^N labelling of arginosuccinate in MR49F cells compared with C4-2B cells (Fig. 7K). We did not detect any labelling of arginine or urea with glutamine-derived nitrogen groups (Fig. 7K). Despite this, our metabolomics analysis detected higher levels of urea in MR49F cells, suggesting that urea cycle nitrogen may be supported by non-glutamine sources of ammonium generated through broader amino acid and nitrogen-containing metabolite catabolism [46].

### H3B-120, a CPS1 inhibitor, slows ENZ-resistant prostate cancer cell proliferation

Based on these findings, we hypothesised that CPS1 constitutes a metabolic vulnerability that can be exploited therapeutically in ENZ-resistant prostate cancer. To test this, we used an inducible CRISPR/Cas9 system to ablate CPS1 in MR49F cells (two independent lines). CPS1 protein abundance was reduced by ~60–65% in the CPS1 KO cell lines (Supplementary Fig. 9E, F). CPS1 depletion did not affect MR49F cell proliferation nor sensitivity to ammonium exposure (Supplementary Fig. 9C, D). Notably, although not quantitative, proteomics showed that MR49F cells had CPS1 levels that were ~90% less than C4-2B, suggesting that a substantially deeper reduction in CPS1 may be required to elicit anti-proliferative effects.

**Fig. 8:**
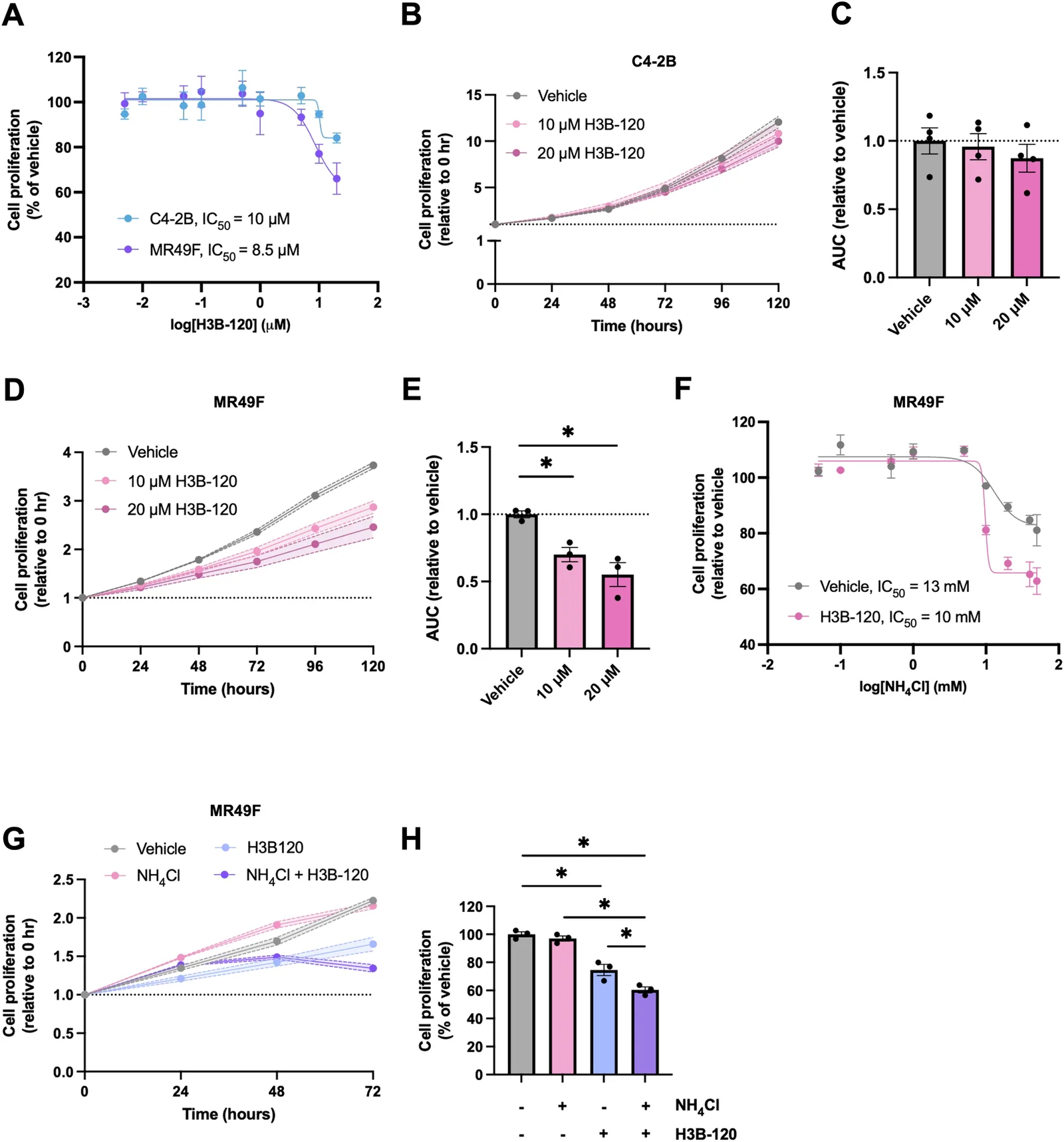
Treatment with the CPS1 inhibitor H3B-120 slows ENZ-resistant prostate cancer cell proliferation. **A** Dose-response curve (non-linear fit) showing percentage cell proliferation of C4-2B (blue) and MR49F (purple) cells at increasing concentrations of H3B-120 (0.05, 0.1, 0.5, 1, 5, 10, 20 ${\rm{\mu }}$M) following 72 h (*n *= 3–4). **B** Cell proliferation of C4-2B cells treated with vehicle or H3B-120 (10 ${\rm{\mu }}$M, 20 ${\rm{\mu }}$M) over five days measured by IncuCyte SX5 live-cell imaging (*n *= 3). **C** Area under curve (AUC, incremental) analysis of growth curves in (**B**). **D** Cell proliferation of MR49F cells treated with vehicle or H3B-120 (10 ${\rm{\mu }}$M, 20 ${\rm{\mu }}$M) over five days measured by IncuCyte SX5 live-cell imaging (*n *= 4). **E** Area under curve (AUC, incremental) analysis of growth curves in (**D**). **F** Dose-response curve (non-linear fit) showing percentage cell proliferation of MR49F cells treated with vehicle or H3B-120 (20 ${\rm{\mu }}$M) at increasing concentrations of NH_4_Cl (0.05, 0.1, 0.5, 1, 5, 10, 20, 40, 50 mM) following 72 h (*n *= 3). **G**, **H** Cell proliferation of MR49F cells treated with vehicle, H3B-120 (20 ${\rm{\mu }}$M), NH_4_Cl (5 mM), or combination over/following 72 h (*n *= 3). Statistical significance was determined using a one-way ANOVA or unpaired Welch’s *t* test, as described in the Methods. *p value < 0.05. Data are presented as mean ± SEM.

As an alternative approach, we used a pharmacological inhibitor of CPS1, H3B-120 [47, 48]. CPS1 inhibition did not affect the proliferation of C4-2B cells (Fig. 8A–C), whereas CPS1 inhibition slowed MR49F cell proliferation at both the low and high dose, by 30% and 45%, respectively (Fig. 8A, D, E). H3B-120 treatment increased the sensitivity of MR49F cells to increasing concentrations of NH_4_Cl (Fig. 8F). CPS1 inhibition alone resulted in a ~30% reduction in cell proliferation, while combining CPS1 inhibition with ammonium slowed MR49F cell proliferation by ~40% (Fig. 8G, H), indicating that H3B-120 suppresses ENZ-resistant cell proliferation, most likely by blocking ammonium detoxification. Collectively, these findings support a potential role for CPS1 in altered ammonium handling in ENZ-resistant prostate cancer cells. Although partial genetic suppression of CPS1 was insufficient to affect cell fitness, pharmacological inhibition reduced proliferation and sensitised MR49F cells to ammonium stress, suggesting that CPS1-mediated nitrogen detoxification may contribute to the survival of ENZ-resistant cells under conditions of ammonium stress.

## Discussion

ENZ and other androgen receptor pathway inhibitors (ARPIs) remain the backbone of therapy for metastatic prostate cancer [49]. Although ENZ slows tumour progression and modestly extends survival, nearly half of patients fail to respond, and resistance inevitably arises in those who initially benefit [49]. Once resistance develops, few effective therapies remain, underscoring the urgent need to understand how prostate cancer cells adapt to survive ENZ treatment.

Multiple AR-centric mechanisms of AR-targeted therapy resistance have been described, including AR mutation, amplification, splice variation, and AR loss. These insights have driven the development of next-generation AR-directed therapies such as PROTAC degraders, non-ligand-binding domain inhibitors, and AR-variant-specific antagonists [50–52]. Despite compelling biological rationale, none have meaningfully improved outcomes for ENZ-resistant prostate cancer. This clinical reality highlights a broader conceptual gap. That is, while AR reactivation remains a defining feature of lethal prostate cancer, resistance is not solely governed by AR biology. Instead, resistance emerges through coordinated cellular adaptations that extend beyond transcriptional rewiring of the androgen axis.

To define the global metabolic alterations that emerge with the acquisition of ENZ resistance, we implemented an integrated multi-omics strategy to comprehensively map changes in metabolite abundance, lipid composition, and protein expression across ENZ-sensitive and ENZ-resistant states. This study expands our understanding of the molecular adaptations associated with ENZ resistance by demonstrating that proteomic and metabolic reprogramming are prominent features of ENZ resistance. Additionally, this metabolic reprogramming is not driven by isolated pathway changes but reflects a broader metabolic program that integrates nitrogen handling, lipid remodelling, and oxidative stress management.

A conceptual insight of this study was the identification of lipid remodelling, rather than ferroptosis resistance alone, as a mechanistic basis of the ENZ-resistant state. Although previous studies have linked ENZ resistance with reduced ferroptotic sensitivity through alterations in glutathione and glutamine metabolism [28], our findings show that established ENZ-resistant cells undergo broad depletion of PUFA-containing lipid species together with suppression of PUFA-synthesis enzymes. This defines a distinct lipidomic mechanism of ferroptosis resistance and suggests that reduced PUFA availability, potentially driven in part by diminished SREBP1-regulated expression, is a functionally important adaptation in acquired ENZ resistance.

Prior work largely supports an AR-linked, pro-tumourigenic role for specific elongases in prostate cancer. For instance, ELOVL7 and ELOVL5 are reported to be elevated in prostate cancer to support lipid elongation across primary and metastatic settings [53, 54]. ELOVL2 has been shown to be upregulated in ENZ-resistant CRPC sublines and promote resistance to ENZ through stabilisation of the AR [55], while ELOVL5 promoted ENZ resistance in neuroendocrine (NE)-like prostate cancer cells [56]. In contrast, our AR-dependent ENZ-resistant model displays broad downregulation of ELOVL5, suggesting that the regulation of individual ELOVLs is context- and stage-dependent and may reflect a shift toward alternative lipid acquisition/remodelling strategies in acquired ENZ resistance.

Tousignant et al. reported that extended ENZ exposure in androgen-responsive prostate cancer cells induces a therapy-tolerant “persister” state characterised by increased membrane PUFA content, attributed predominantly to increased uptake of exogenous lipids [33]. Importantly, PUFA-enriched membranes sensitised these “persister” cells to GPX4 inhibition and ferroptosis, suggesting a transient therapeutic opportunity to exploit early ENZ adaptation by co-targeting lipid handling pathways or modulating extracellular PUFA/antioxidant availability. Although prolonged ENZ exposure has been associated with increased lipid peroxidation, we did not detect a significant increase in C4-2B cells after two or five days of treatment, which may reflect the shorter treatment duration used in our experiments.

In contrast to the PUFA-enriched persister state, the ENZ-resistant phenotype analysed in the present study exhibits depletion of PUFA-containing lipids consistent with diminished susceptibility to lipid peroxidation and GPX4 inhibition. This supports a model in which lipid rewiring evolves over time with the development of ENZ resistance, from an early PUFA-enriched “persister” phase that is ferroptosis-prone, to a later acquired resistance state that actively limits PUFA availability to evade ferroptotic stress. Overall, these findings contribute to a growing body of work exploring the role of lipid peroxidation stress and ferroptosis in ENZ response and resistance in prostate cancer [28, 29, 33, 57].

Remarkably, in our study, supplementing resistant cells with exogenous PUFAs or activation of LXR restored sensitivity of ENZ-resistant cells to lipid peroxidation and GPX4 inhibition-induced ferroptosis, demonstrating that ferroptosis resistance is both reversible and metabolically governed in ENZ-resistant prostate cancer. The different effects of linoleate in the lipid-peroxidation and proliferation assays may reflect their distinct experimental durations. Lipid peroxidation was measured after 2 h of RSL3 treatment, whereas proliferation was assessed over 72 h, potentially allowing linoleate to be elongated and desaturated into more highly unsaturated downstream fatty acids during the longer assay. We attempted to measure lipid peroxidation following longer RSL3 exposure, however, extensive cell death prevented reliable flow-cytometric gating and quantification. Moreover, LXR activation has pleiotropic effects on lipid metabolism and other cellular pathways, and these experiments therefore do not establish that the increased ferroptotic sensitivity was mediated exclusively through SREBP1. In a complementary report, PUFA supplementation with DHA increased intracellular ROS and lipid peroxidation in prostate cancer cells and suppressed tumour growth [58]. Together, these data suggest that PUFA availability shapes the coupling between oxidative stress and lipid peroxidation, and that this coupling during ENZ adaptation, creates a metabolically imposed but reversible brake on GPX4-inhibitor-induced ferroptosis.

A central metabolic adaptation observed in ENZ-resistant cells was enhanced ammonium tolerance driven by activation of CPS1. ENZ-resistant cells upregulated CPS1, accumulated N-acetyl-glutamate, and displayed resilience to supraphysiological ammonium exposure. Yet, glutamine tracing revealed minimal flux of glutamine-derived nitrogen into downstream urea-cycle intermediates, potentially reflecting non-canonical re-routing of carbamoyl phosphate to nucleotide synthesis [59] or nitric oxide synthesis [60], rather than classical ureagenesis. Importantly, our tracer design specifically followed glutamine as the sole nitrogen donor and therefore does not exclude the possibility that other nitrogen sources (e.g., additional amino acids or free ammonium) contribute to nitrogen handling and replenishment of these intermediates in ENZ-resistant cells. Future studies should therefore extend this work by directly tracing nitrogen from ^15^N-labelled NH_4_^+^ over different time points (and complementary amino-acid tracers) to more comprehensively map the sources and destinations of intracellular nitrogen under ENZ-resistant conditions.

Notably, partial genetic depletion of CPS1 and treatment with the CPS1 inhibitor H3B-120 produced discordant phenotypes. CPS1 knockdown reduced protein abundance by ~60% but did not significantly impair cell viability, whereas H3B-120 reduced proliferation and sensitised MR49F cells to ammonium stress. This difference may reflect sufficient residual CPS1 activity following incomplete depletion, although potential off-target effects of H3B-120 cannot be excluded. Accordingly, these findings support CPS1 as a candidate contributor to altered ammonium handling rather than establishing a definitive CPS1 dependency. This work uncovers a previously understudied metabolic adaptation in prostate cancer, and aligns with the activation of a CPS1-mediated stress tolerance that has been identified in other tumour types [59, 61, 62]. Bringing together our findings, glutamine-derived ammonium has been shown to activate SREBP1 in cancer cells [63, 64], providing a potential causal link between CPS1 activity and PUFA synthesis that are features of ENZ-resistant prostate cancer.

Metabolic tracing, lipidomic, metabolomic and proteomic assessment of matched ENZ-sensitive and ENZ-resistant prostate cancer cells revealed extensive, interconnected rewiring of carbon metabolism, lipid turnover, and redox regulation. Importantly, these adaptations appear to improve the ability of resistant cells to withstand metabolic and oxidative stress, enabling persistence despite AR pathway suppression. This integrative view counters reductionist explanations that single molecular events, such as AR mutations or splice variants, drive ENZ resistance. Instead, our findings show that ENZ resistance is likely to emerge through coordinated alterations across several distinct metabolic pathways that promote cellular resilience, allowing prostate cancer cells to overcome clinical treatment.

Our study has limitations. Immortalised cell lines lack the stromal interactions, extracellular matrix, and intra-tumoural heterogeneity of clinical prostate cancer [65]. Future studies should validate these findings in patient-derived xenografts and organoid systems that better recapitulate in vivo nutrient gradients and microenvironmental constraints. Furthermore, only two ENZ-sensitive lines and one ENZ-resistant line were profiled, potentially limiting generalisability. Notably, while other matched castration-resistant and ENZ-resistant model pairs are available, the MR49F cell line is one of the few that remains resistant even at high doses of ENZ. This partially mitigates the limitation of relying on a single model pair, while also highlighting the need for additional validated models that reproduce this degree of resistance. Direct clinical validation was limited by the absence of matched tumour proteomic, metabolomic and lipidomic datasets collected before and after the acquisition of ENZ resistance. Available clinical studies are predominantly restricted to tumour transcriptomics or plasma-based profiling and therefore cannot directly determine whether the integrated intracellular metabolic phenotype identified here is recapitulated in patient tumours. Future studies using longitudinal tumour specimens and patient-derived models will be required to establish the clinical relevance of these findings. Lastly, the omics approaches utilised in this study provide static snapshots of abundance rather than dynamic flux for most pathways. Broader metabolic tracing beyond ^14^C-metabolic tracing is needed to fully define causal relationships between enzyme regulation and pathway activity.

Taken together, our data indicate that ENZ-resistant prostate cancer cells acquire a distinct metabolic profile marked by increased non-canonical nitrogen handling and PUFA depletion. By demonstrating that ferroptosis resistance is metabolically reversible, and that CPS1-dependent nitrogen handling supports tolerance to ammonium stress, this study identifies two distinct metabolic adaptations that protect ENZ-resistant prostate cancer cells from lethal stress. These findings provide a framework for exploiting cancer metabolism to re-engage cell stress pathways in ENZ-resistant prostate cancer.

## Methods

### Cell culture

C4-2B (RRID:CVCL 4784) cells were obtained from ATCC. V16D cells were provided by Dr Luke Selth. MR49F cells were provided by Dr Amina Zoubeidi. All cell lines were maintained in RPMI-1640 medium supplemented with GlutaMAX (Gibco, 61870036), 10% fetal bovine serum (FBS, AusBiotech), and 1% penicillin-streptomycin (Gibco, 15140122). Enzalutamide (ENZ; 10 μM, Selleckchem) was added to the culture medium for MR49F cells. Cultures were maintained at 37 °C in a humidified incubator with 5% CO₂. Cell line identity was confirmed by short tandem repeat profiling using the Promega GenePrint 10 system (Australian Genome Research Facility), and all cultures were routinely tested for mycoplasma contamination.

### ^14^C-labelled glucose, glutamine, palmitate, and lactate tracing

C4-2B and MR49F cells were incubated for 4 h at 37 °C in 500 µL low-glucose DMEM (Gibco, catalog no. 10567022) containing 5.5 mM glucose and 4 mM glutamine, supplemented with 2% ultra-low fatty acid bovine serum albumin (BSA; Bovogen, BSAS-AU), 0.5 mM palmitate (Sigma, catalog no. P0500), 2 mM sodium lactate, and 1 mM L-carnitine. For metabolic tracing experiments, media were additionally supplemented with either ^14^C-labelled glucose (1 µCi/mL; PerkinElmer, catalog no. NEC042A001MC), ^14^C-labelled glutamine (1 µCi/mL; PerkinElmer, catalog no. NEC451050UC), ^14^C-labelled palmitic acid (1 µCi/mL; PerkinElmer, catalog no. NEC075H250UC), or ^14^C-labelled lactic acid (0.1 µCi/mL; PerkinElmer, catalog no. NEC599050UC, lot no. 2396656).

Complete substrate oxidation was assessed by trapping released ^14^CO_2_ in 1 M NaOH following acidification of the incubation medium with 1 M HClO (1:1, v/v). Trapped radioactivity was quantified by scintillation counting. Following incubation, cells were washed with Dulbecco’s phosphate-buffered saline (DPBS; Gibco, catalog no. 14190250) and lysed in 300 µL DPBS containing 0.01% Triton X-100. Methanol/chloroform (2:1, v/v; 1.8 mL) was added to the lysates to extract metabolites and lipids. The aqueous phase was collected for scintillation counting to quantify incorporation into aqueous soluble metabolites. The organic phase was dried in a continuous stream of nitrogen at 40 °C and reconstituted in 30 µL methanol/chloroform (2:1, v/v) containing lipid standards including cholesterol, triglyceride, oleate, diglyceride, ceramide, and phospholipid.

### Thin-layer chromatography

Incorporation of radiolabelled substrate into complex lipids was assessed by thin-layer chromatography (TLC). Reconstituted lipid extracts were spotted onto 250 µm glass-backed silica gel plates (Thomas Scientific). Lipids were separated using a three-step solvent system. First, plates were developed in chloroform/methanol/water (65:25:4, v/v/v) until the solvent front reached 50% of the plate length. Plates were then air dried and developed in hexane/diethyl ether/acetic acid (75:35:1, v/v/v) until the solvent front reached 90% of the plate length. After air drying, plates were developed a second time in the same resolving solution. Plates were dried, sprayed with dichlorofluorescein (Sigma, catalog no. D6665), and visualised under UV light. Lipid bands corresponding to standards were scraped and radioactivity was quantified by scintillation counting. For glucose, glutamine, palmitate, and lactate tracing experiments, substrate uptake was calculated as the sum of complete oxidation, incorporation into aqueous soluble metabolites, and incorporation into complex lipids. The aqueous-soluble metabolite fraction was not included in this calculation for glucose and lactate because the recovered radioactive signal was too low for reliable quantification. Metabolic rates were normalised to protein concentration, determined using the Pierce BCA Protein Assay (Thermo Fisher Scientific, catalog no. 23225) according to the manufacturer’s instructions.

### Protein extraction and preparation for LC–MS/MS (proteomics)

Cells were rinsed twice with ice-cold DPBS and lysed in 300 µL of buffer containing 0.05 M triethylammonium bicarbonate (TEAB), 5% sodium dodecyl sulfate (SDS), and mass spectrometry (MS)-grade water. Lysates were snap-frozen on dry ice and stored at −80 °C. Protein solubilisation, reduction, alkylation, and clean-up were performed using S-Trap micro columns (Protifi) according to the manufacturer’s protocol. Proteins were digested overnight at 37 °C with sequencing-grade trypsin (1 µg per 100 µg protein) and Lys-C (0.5 µg per 100 µg protein). Peptides were desalted using in-house packed SDB-RPS StageTips (Sigma-Aldrich, #66886-U), washed with acidified isopropanol and aqueous acetonitrile (ACN)/ trifluoroacetic acid (TFA) buffers, and eluted with 80% ACN containing ammonium hydroxide, adapted from Blazev et al. [66]. Samples were dried by SpeedVac at 45 °C and resuspended in ACN with 0.05% formic acid.

### Data-independent LC-MS/MS acquisition (proteomics)

Peptides were analysed on an Ultimate 3000 RSLC nanoLC system (Thermo Fisher Scientific) equipped with an Acclaim PepMap RSLC analytical column (C18, 75 µm × 50 cm, 100 Å) and a PepMap trap column (75 µm × 2 cm, 100 Å), both maintained at 50 °C. Solvent A consisted of 0.1% formic acid and 5% dimethyl sulfoxide (DMSO) in water; solvent B consisted of 0.1% formic acid and 5% DMSO in ACN. Trap loading was performed at 6 µL/min for 6 min using 3% ACN/0.05% TFA, followed by gradient elution at 300 nL/min: 3–4% B (1 min), 4–25% B (75 min), 25–40% B (4 min), 40–80% B (1 min), hold at 80% B (3 min), and re-equilibration for 4.9 min.

Data-independent acquisition was performed on an Orbitrap Exploris 480 mass spectrometer (Thermo Fisher Scientific) operated with a spray voltage of 1.9 kV, ion funnel RF of 50%, and capillary temperature of 275 °C. Full MS scans were acquired from m/z 350–1400 at 120,000 resolution with AGC target 250% and 50 ms maximum injection time. DIA consisted of 49 windows (13.7 m/z width) spanning m/z 360–1046 with MS/MS scans acquired at 30,000 resolution, AGC 1 × 10⁶, 55 ms maximum injection time, and NCE 30%.

### DIA data processing (proteomics)

DIA files were analysed using Spectronaut v16 with default direct-DIA settings. Enzyme specificity was set to trypsin with ≤2 missed cleavages. Carbamidomethyl-cysteine was specified as a fixed modification and methionine oxidation and N-terminal acetylation as variable modifications. Mass tolerances for MS1 and MS2 spectra were determined by Spectronaut’s dynamic calibration algorithm. Spectra were searched against the UniProt human reference proteome. False discovery rates were controlled at 1% at the PSM, peptide, and protein levels. MaxLFQ was enabled for label-free quantification, and no imputation was applied.

### Lipid/metabolite extraction and preparation for LC–MS/MS

Culture medium was aspirated, and cells were washed twice with ice-cold DPBS while maintained on ice. Plates were then transferred to dry ice, and 1 mL of 100% MS-grade methanol (pre-cooled at −80 °C for ≥1 h) was added directly to each plate. Cells were scraped immediately in methanol on dry ice and collected. Samples were sonicated for 15 min at 4 °C, centrifuged, and the clarified methanolic lysates (1 mL) were stored at −80 °C.

Cell metabolite extraction method was adapted from the SIMPLEX protocol [67]. Briefly, 225 µL of cold methanol was added to dried cell lysates, followed by vortexing for 20 s. Next, 750 µL of cold MTBE was added, and samples were incubated for 1 h at 4 °C with agitation (1200 rpm). Phase separation was induced by adding 188 µL of water containing 0.1% ammonium acetate. Samples were then centrifuged at 13,000 × *g* for 12 min at 4 °C. The upper organic phase (lipid-containing fraction; ~700 µL) was carefully transferred to a 1.5 mL Eppendorf tube. The remaining aqueous phase was centrifuged again under the same conditions, and 250 µL of the lower phase (metabolite-containing fraction) was collected into a separate 1.5 mL Eppendorf tube. Both fractions were dried using a miVAC centrifugal evaporator.

The organic fraction was reconstituted in 160 µL of 90:10 isopropanol:ACN. The aqueous fraction was reconstituted in 90% methanol. Samples were vortexed for 10–15 s, sonicated for 15 min, and centrifuged at 12,000 rpm at 4 °C for 10 min. The supernatants were then collected. For each cell line, pooled QC samples (technical QC, TQC, and response QC, RQC) were prepared by taking 20 µL from each sample. Three process blank (PBLK) samples were also prepared using the same extraction protocol described above, but without cell lysates. Finally, 50 µL of the samples were transferred to autosampler vials for LC-MS analysis.

### Untargeted lipidomics data acquisition

The LC-MS system used comprised of an Agilent 1290 HPLC (Agilent, USA) and a TripleTOF^TM^ 6600 equipped with a DuoSpray^TM^ ion source (SCIEX, USA). Automated mass calibration was performed periodically after every ten injections. Chromatography was performed on a ZORBAX RRHD Eclipse Plus C18, 1.8 μm, 2.1 × 50 mm column (Agilent, USA). Mobile phase A consisted of water/ACN (60:40, v/v) with 10 mM ammonium formate, and mobile phase B consisted of IPA/ACN (90:10, v/v) with 10 mM ammonium formate. The flow rate was 0.4 mL/min, and the column temperature was set at 40 °C. The gradient was programmed from 80% A to 40% A at 2 min, decreased to 0% A by 12 min, held until 14 min, returned to 80% A at 14.1 min, and maintained until the end of the run. An injection volume of 2 µL was used for cell extract samples, PBLK, and QC samples. For RQC samples, six varying injection volumes corresponding to sample loads of 25–150% was used in SWATH acquisition mode for response linearity determination.

For MS analysis, DDA was applied to QC samples for compound identification and SWATH was applied to study samples for semi-quantification. Both positive and negative ionisation modes were used. Ion source parameters for both DDA and SWATH were as follows: ion source gas 1 (N2) 50 psi; ion source gas 2 (N2) 60 psi; curtain gas (N2) 35 psi; CAD gas (N2) 7 psi; source temperature 500 °C; spray voltage +5500 V (positive mode) and −4500 V (negative mode); declustering potential ±80 V. For both acquisition modes, collision energy 10 V was used for TOF-MS and 45 V was used for TOF-MSMS for fragmentation. The mass range was 300–1000 m/z for TOF-MS and 50–1000 m/z for TOF-MSMS experiments. In DDA experiment, accumulation time was 0.1 s for TOF-MS and 0.05 s for TOF-MSMS. Maximum candidate ion was set to 15, with former candidate ions excluded for 2 s after 2 occurrences. In the SWATH acquisition, accumulation time was 0.05 s in TOF-MS and fixed windows were applied in TOF-MSMS experiment with window widths of 10 Da and accumulation time of 0.025 per isolation window (70 isolation windows).

### Untargeted metabolomics data acquisition

The LC-MS system used comprised of an Agilent 1290 HPLC (Agilent, USA) and a TripleTOF^TM^ 6600 equipped with a DuoSpray^TM^ ion source (SCIEX, USA). Automated mass calibration was performed periodically after every ten injections. Chromatography was performed on a SeQuant® ZIC®-cHILIC, 3 µm, 100 Å 100 × 2.1 mm column (Merck, Germany). Mobile phase A consisted of 10 mM ammonium formate in water. Mobile phase A was water with 10 mM ammonium formate and mobile phase B was acetonitrile with 0.1% formic acid. Flow rate was 0.250 ml/min and column temperature was 40 °C. The LC gradient started at 3% A, increased to 30% A from 1 to 12 min, then to 90% A from 12 to 15 min, remained at 90% until 18.5 min, and returned to 3%, holding until the end of the 24-min run. An injection volume of 6 µL was used for cell extract samples, PBLK, and QC samples. For RQC samples, six varying injection volumes corresponding to sample loads of 25–150% was used in SWATH acquisition mode for response linearity determination.

For MS analysis, DDA was applied to QC samples for compound identification and SWATH was applied to study samples for semi-quantification. Ion source parameters for both DDA and SWATH were as follows: ion source gas 1 (N2) 50 psi; ion source gas 2 (N2) 60 psi; curtain gas (N2) 35 psi; CAD gas (N2) 7 psi; source temperature 500 °C; spray voltage +5500 V (positive mode) and −4500 V (negative mode); declustering potential ±80 V. For both acquisition modes, collision energy 10 V was used for TOF-MS and 30 V was used for TOF-MSMS for fragmentation. The mass range was 55–1000 m/z for TOF-MS and 40–1000 m/z for TOF-MSMS experiments. In DDA experiment, accumulation time was 0.1 s for TOF-MS and 0.05 s for TOF-MSMS. Maximum candidate ion was set to 15, with former candidate ions excluded for 2 s after 2 occurrences. In the SWATH acquisition, accumulation time was 0.05 s in TOF-MS and fixed windows were applied in TOF-MSMS experiment with window widths of 20 Da and accumulation time of 0.02 per isolation window (45 isolation windows).

### Data processing

Acquired data files were converted to mzML file format. Data processing was performed using MetaboKit [68]. Metabolite identification was performed using a curated pipeline that integrates MS2 spectral matching and MS1-level precursor matching. For spectral matching against reference libraries, metabolites were annotated to peak features by selecting the top-scoring spectrum with a minimum cosine similarity score of 0.5. The MetaboKit output file containing library-matched features was filtered according to detected frequency (Freq) ≥ 0.5 for MS1-level IDs and 0.2 for MS2-level IDs, signal-to-blank ratio (S/BLK) ≥ 5, and median peak area >1000. The curated list of DDA features was exported as an MSP format spectral library and used for SWATH data processing to generate an output file containing peak areas of SWATH MS1 and MS2 features. SWATH data were filtered using data from TQC, RQC, and PBLK samples (Freq ≥0.5 (MS1-level ID) and 0.25 (MS2-level ID), S/BLK ≥ 3, coefficient of variation ≤30, score ≥0.8 and linearity (RSQ) ≥ 0.7) to yield the final list of metabolite features. Data were subsequently normalized using a mean-SD normalisation approach for downstream analysis.

### Metabolite extraction and derivatisation for GC–MS

Intracellular metabolites were extracted using a monophasic methanol-chloroform protocol. To adherent cells, 600 µL of pre-chilled 9:1 MeOH/chloroform containing 0.4 nmol ¹³C₅¹⁵N-valine and 0.4 nmol ¹³C₆-sorbitol was added. Cells were scraped for 30 s and lysates transferred to microcentrifuge tubes, vortexed, and incubated at 4 °C for 10 min with agitation (950 rpm). Samples were centrifuged at 16×000 × *g* for 10 min at 4 °C. Supernatants were collected, pooled to generate a biological QC, and evaporated to dryness at 30 °C (CHRIST RVC 2-33 CDplus). Residual moisture was removed by addition and re-evaporation of 50 µL 100% methanol.

Derivatisation was performed online using a Shimadzu AOC6000 robot. Dried extracts were incubated with 25 µL methoxyamine hydrochloride (30 mg/mL in pyridine) for 2 h at 37 °C, followed by 25 µL BSTFA + 1% TMCS for 1 h at 37 °C. Samples were equilibrated at room temperature for 1 h before split injection (1:10).

### GC–MS/MS acquisition and data processing

Metabolite analysis was performed using a Shimadzu 2030 GC coupled to a TQ8050NX triple quadrupole mass spectrometer operated in EI mode (−70 eV). Chromatography was performed on a DB-5 column (30 m × 0.25 mm, 1 µm film). The inlet, transfer line, and ion source temperatures were 280 °C, 280 °C, and 200 °C, respectively. The oven program was: 100 °C (4 min), ramp to 320 °C at 10 °C/min, hold for 11 min. Helium was used as the carrier gas (1 mL/min), and argon served as the collision gas. Approximately 625 MRM targets (representing ~420 metabolites) were monitored using the Shimadzu Smart Metabolite Database. Samples were analysed in randomised order with PBQC injections every 5 runs. Peak integration was performed in LabSolutions Insight and manually curated.

### Data pre-processing, filtering, and normalisation

Raw data were imported into R. Metadata columns were removed, and missing values were replaced with zeros. Low-abundance proteins were excluded if >20% of values fell below the 1st percentile of all non-zero intensities. Remaining values were offset by adding the 1st percentile constant to stabilise downstream analysis.

For lipidomic and metabolomic datasets, samples were stratified by ionisation mode, log₂-transformed, centred, and scaled within each mode. Scaled values were exponentiated (2ˣ) and recombined for downstream analyses. Differential abundance analysis was performed and false discovery rate (FDR) was calculated using unpaired two-tailed Welch’s *t* tests with multiple-testing correction using the Benjamini–Hochberg (BH) method via the base stats package. A false discovery rate (FDR) < 0.05 was considered statistically significant.

### Principal component analysis (PCA)

Filtered datasets were log₂-transformed and scaled prior to dimensionality reduction. PCA was performed using the prcomp() function in R with centring and scaling enabled. Data visualisation was performed using ggplot2.

### Protein set enrichment analysis (PSEA)

Gene symbols were mapped to Entrez IDs and ranked by directional significance: Rank = sign(log₂FC) × -log₁₀(FDR). PSEA was performed using the GSEA() function in clusterProfiler with BH correction. Selected pathways were visualised using ggplot2 and gseaplot2().

### Transcription factor activity analysis

Transcription factor (TF) activity was inferred from proteomic differential expression data using the *decoupleR* package in R. A human TF-target interaction network was obtained from CollecTRI through the *OmnipathR* package, with protein complexes retained as single entities. Fold-change values were converted to log2 scale, and a signed gene-level statistic was generated by combining the direction of change with the adjusted *p* value. These statistics were used as input for the univariate linear model method implemented in *decoupleR* to estimate TF activity scores. Only TFs with at least five target genes represented in the dataset were included in the analysis. TFs were ranked according to their inferred activity scores, and the top-ranked TFs were visualised using bar plots generated with *ggplot2*.

### Lipid class aggregation

High-confidence MS/MS-annotated lipids were retained, and duplicate features were collapsed by selecting the entry with the highest mean abundance. Lipid classes were extracted from compound names, and median class abundances were calculated. Class values were normalised to the mean of control samples and log₂-transformed. Heatmaps were generated using *ComplexHeatmap* with Euclidean distance and average linkage clustering. *Lipid saturation analysis* Saturation level (number of double bonds) was extracted from lipid names. Lipids were grouped by saturation level (0-9), and median abundances were computed per sample. Values were normalised to controls and exported for downstream interpretation. Saturation values were normalised to the mean of control samples and log₂-transformed. Heatmaps were generated using *ComplexHeatmap* with Euclidean distance and average linkage clustering.

### Quantitative real-time PCR

RNA was extracted using TRI Reagent (Sigma-Aldrich, T9424) with phase separation using 100 µL chloroform. After centrifugation (12 000 g, 15 min, 4 °C), the aqueous phase was precipitated in 500 µL isopropanol, washed in 100% ethanol, and resuspended in 30 µL nuclease-free water. RNA concentration and purity were assessed using a SpectroSTAR Nano system (BioRad).

cDNA was synthesised from 1 µg RNA using the iScript cDNA Synthesis Kit (BioRad, 1708891). qPCR reactions contained 2 µL cDNA, 5 µL SYBR Green PCR Master Mix (Qiagen, 208054), and 1 µL primer mix (sequences in Supplementary Table 2). Cycling was performed on a CFX384 Touch Real-Time PCR system (BioRad). Gene expression was normalised to the geometric mean of *ACTB* and *RPLP0* and analysed by the ^ΔΔ^Ct method (sequences in Supplementary Table 2).

### Fatty acid supplementation

Cells were seeded and allowed to adhere overnight. Media were replaced with RPMI containing 1% BSA and 10% FBS, supplemented with either vehicle (ethanol) or 10 µM fatty acids: palmitic acid (Sigma, P0500), linoleic acid (Sigma, L1376), arachidonic acid (Sigma, A3611), or docosahexaenoic acid (Sigma, D2534).

### IncuCyte proliferation assays

Cells were plated in 96-well plates (C4-2B: 500 cells/well; MR49F: 1000 cells/well). For fatty acid and LXR agonist experiments, cells were pre-treated with fatty acids or GW3965 (MedChemExpress, HY-10627) for 16 hours prior to treatment with RSL3 (Selleckchem, #S8155). For CPS1 inhibition and ammonium-stress experiments, cells were treated with H3B-120 (Merck, SML3007) and/or ammonium chloride (NH₄Cl; AnalaR, VWR International, 100173D) at the indicated concentrations and for the durations specified in the figure legends. Treatments were applied and proliferation was monitored on an IncuCyte SX5 system (Sartorius) using 10× phase-contrast imaging. Five fields per well were collected every 48 h. Confluency was quantified using IncuCyte analysis software and normalised to 0 h.

### Flow cytometry

Cells were incubated with C11-BODIPY 581/591 (Invitrogen; 1:1 000) or MitoPerOx (Abcam, ab146820; 1:1 000) diluted in DPBS for 30 min at 37 °C. After trypsinisation, cells were washed twice, filtered, and analysed on a CytoFLEX LX or CytoFLEX S (Beckman Coulter). Gating strategy described in Supplementary Fig. 9. Oxidised C11-BODIPY was excited at 488 nm (525/40 nm emission) and the reduced form at 561 nm (610/20 nm). Median fluorescence intensity was calculated using FlowJo.

### Immunoblotting

Cells were lysed in RIPA buffer (150 mM NaCl, 1% NP-40, 65 mM Tris-HCl pH 7.4, 0.5% sodium deoxycholate, 0.1% SDS, 10% glycerol) containing protease (cOmplete, Roche) and phosphatase inhibitors (PhosSTOP, Roche). Lysates were sheared through a fine-gauge needle and incubated for 1 h at 4 °C before clarification (12 000 g, 10 min). Protein concentration was measured by BCA assay (Thermo Fisher, 23225). Samples were denatured in Laemmli buffer containing dithiothreitol (DTT) at 65 °C for 15 min.

Equal protein amounts were resolved by SDS-PAGE (10% Mini-PROTEAN TGX, Bio-Rad), activated using stain-free imaging (ChemiDoc MP and ImageLab software Version 4.1, BioRad Laboratories), and transferred to PVDF membranes using a Trans-Blot Turbo system. Membranes were blocked in 5% skim milk/TBST or 5% BSA/TBST and incubated overnight at 4 °C with anti-SREBP1 (BD Pharmingen, 557036; 1:500) or anti-CPS1 (Abcam, ab45956; 1:1 000). After washing, membranes were incubated with HRP-conjugated secondary antibodies (Cytiva, NA9310V; 1:10 000) for 2 h at room temperature. Chemiluminescence was detected using Immobilon Western HRP substrate (Millipore, WBULS0100) on a ChemiDoc MP. Quantified data are presented as the density of the immunoreactive band relative to total protein loading for that specific lane.

### Lipidomics for PUFA quantification

MR49F cells were seeded and treated with GW3965 for 24 and 48 hours. Cells were collected in 500 µL ice-cold PBS and snap frozen. Lipids were extracted by sequential addition of 120 µL Milli-Q water, 420 µL methanol containing 0.01% BHT, and 260 µL chloroform containing 0.01% BHT, with brief vortexing after each addition, producing a monophasic solvent system with a final H₂O:CHCl₃:MeOH ratio of 0.74:1:2. Samples were then vortexed vigorously for 1 min, incubated at room temperature for 30 min with shaking at 1,400 rpm, and centrifuged at 14,000 rpm for 15 min. The supernatant was carefully transferred to a fresh 2 mL tube without disturbing the pellet. The pellet was re-extracted with 100 µL Milli-Q water and 400 µL chloroform:MeOH (1:2, v/v, containing 0.01% BHT), vortexed, incubated at room temperature for 30 min with shaking at 1 000 rpm, and centrifuged again at 14 000 rpm for 15 min. The second supernatant was pooled with the first, while the residual pellet was retained for total protein quantification. Combined extracts were dried using a rotary vacuum evaporator or under a stream of nitrogen and resuspended in chloroform:methanol (1:9, v/v, containing 0.01% BHT), vortexed for 20 s, and centrifuged at 14,000 × g for 15 min to remove residual protein or salt complexes. The clarified supernatant was then transferred to glass LC-MS vials for analysis.

Samples were analysed by ultrahigh performance liquid chromatography (UHPLC) coupled to tandem mass spectrometry (MS/MS) employing a Vanquish UHPLC linked to an Orbitrap Fusion Lumos mass spectrometer (Thermo Fisher Scientific, San Jose, CA, USA), with separate runs in positive and negative ion polarities. Solvent A was 6/4 (v/v) acetonitrile/water with 5 µM medronic acid and solvent B was 9/1 (v/v) isopropanol/acetonitrile. Both solvents A and B contained 10 mM ammonium acetate. 10 μL of each sample was injected into an Acquity UPLC HSS T3 C18 column (1 ×150 mm, 1.8 µm; Waters, Milford, MA, USA) at 50 °C at a flow rate of 60 μL/min. The solvent timetable was as follows [time (min), %B]: [0, 3], [5, 3], [10, 70], [26, 99], [29, 99], [29.1, 3], [33, 3]. All MS analysis were performed using an electrospray ionisation source (3.5 kV in positive- and 3.0 kV in negative ionisation-mode). In both polarities, the flow rates of sheath, auxiliary and sweep gases were 25 and 5 and 0 arbitrary unit(s), respectively. The ion transfer tube and vaporizer temperatures were maintained at 300 °C and 150 °C, respectively, and the ion funnel RF level was set at 50%. From 5 to 33 min, top speed data-dependent scan with a cycle time of 1 s was used. Within each cycle, a full-scan MS-spectra were acquired firstly in the Orbitrap at a mass resolving power of 120 000 (at *m/z* 200) across an *m/z* range of 200–2 000. The automatic gain control (AGC) target and maximum injection time were set to ‘standard’ and ‘auto’, respectively. Higher-energy collisional dissociation (HCD)-MS/MS spectra were collected at a mass resolving power of 15,000 (at *m/z* 200), a normalized collision energy (NCE) of 27% at positive mode and 30% at negative mode, an *m/z* isolation window of 1. For the improved structural characterisation of glycerophosphocholine (PC) lipid cations, a data-dependent product ion (*m/z* 184.0733)-triggered collision-induced dissociation (CID)-MS/MS scan was performed in the cycle using a q-value of 0.25 and an NCE of 30%, with other settings being the same as that for HCD-MS/MS. For the improved structural characterisation of triacylglycerol (TG) lipid cations, the fatty acid + NH3 neutral loss product ions observed by HCD-MS/MS were used to trigger the acquisition of the top-3 data-dependent ion trap CID-MS3 scans in the cycle using a q-value of 0.25 and an NCE of 30%, with other settings being the same as that for HCD-MS/MS. Dynamic exclusion of 15 seconds was enabled and only ions with charge state of 1–3 were selected for fragmentation.

Raw LC-MS/MS data were processed in MS-DIAL for peak detection, alignment and lipid annotation. PC lipid peaks were manually inspected and curated to confirm peak integration, retention time alignment and MS/MS spectral support. Exported PC peak areas were normalised to the relevant internal standard and subsequently normalised to total protein content from the residual cell pellet. Protein-normalised PC abundances were used for downstream PUFA quantification.

### Generation of inducible CPS1 knockout cell lines

Inducible CPS1 knockout cell lines were generated by transducing tet-pLKO-sgRNA-puro (Addgene #104321) into Cas9-expressing MR49F cells, previously established using the FUCas9Cherry vector (Addgene #70182). sgRNA sequences targeting CPS1 were designed using the CHOPCHOP algorithm. Complementary oligonucleotides (sequences in Supplementary Table 3) were synthesised with appropriate overhangs for cloning into the AjuI restriction site. Oligos were resuspended to 10 μM, annealed by heating to 95 °C for 5 min, and gradually cooled to 25 °C at 2.5 °C/min.

Annealed oligos were diluted (1:200) and ligated into AjuI-digested tet-pLKO-sgRNA-puro vector using T4 DNA ligase. Ligation reactions (10 μl total volume) contained 50 ng digested backbone, 1 μl annealed oligo, 1 μl 10× ligase buffer, and 0.5 μl T4 DNA ligase (NEB), and were incubated at room temperature for 10 min. Constructs were transformed into NEB Stable competent cells, and individual colonies were expanded overnight. Plasmids were purified (PureYield Miniprep, Promega) and verified by Sanger sequencing (AGRF) using the primer 5′-GTTTCAGACCCACCTCCCAAC-3′.

Lentiviral particles were produced using a third-generation packaging system by transient transfection of HEK-293T cells with pMDLg/pRRE (Addgene #12251), pRSV-REV (Addgene #12253), pVSV-G (Addgene #14888), and the transfer plasmid, using polyethyleneimine (PEI). Viral supernatant was collected 48 h post-transfection, filtered, and used for transduction.

For lentiviral transduction, HEK-293T cells were transfected with packaging plasmids and tet-pLKO-sgRNA-puro constructs to generate viral particles. Cas9-expressing MR49F cells were transduced with viral supernatant in the presence of polybrene (8 μg/ml) overnight. Cells were then cultured in fresh media and selected with puromycin (2 μg/ml) for 3 days, followed by expansion under continued selection for one additional passage. sgRNA expression was induced by treatment with doxycycline (1 μg/ml) for five days prior to downstream experiments.

### Statistical analysis

All cell line experiments were performed with a minimum of three independent biological replicates unless otherwise stated. All statistical analyses were performed using standard parametric tests as follows. Comparisons between two independent groups were assessed using two-tailed Welch’s *t* test. Comparisons of primary interest were MR49F versus C4-2B and MR49F versus V16D. Normality was assessed where appropriate using Shapiro–Wilk test. For two-group comparisons derived from multi-omic datasets, *P* values were corrected for multiple testing using the Benjamini–Hochberg procedure, and features were considered significant at a false discovery rate (FDR) < 0.05. Comparisons involving three or more groups were assessed using one-way analysis of variance (ANOVA), followed by an appropriate post hoc multiple comparisons where applicable. Unless otherwise stated, results are presented as mean ± SEM, and statistical significance was defined as *P* < 0.05. Custom code used for data processing, statistical analysis and figure generation is available from the corresponding author upon reasonable request.

## Supplementary information

Supplementary figures

Supplementary data

Uncropped western blots

## Data Availability

All data generated or analysed during this study are included in this published article and its Supplementary Information files.
