# Supplementary material for: Multi-omic profiling reveals metabolic vulnerabilities in enzalutamide resistant prostate cancer
Source: Cell Death Discov. 2026 Sep 9;12:431. doi: 10.1038/s41420-026-03332-3 (PMC13720084; doi:10.1038/s41420-026-03332-3)
Supplement: Supplementary file 1 [file 41420_2026_3332_MOESM1_ESM.pdf]

Supplementary Figures

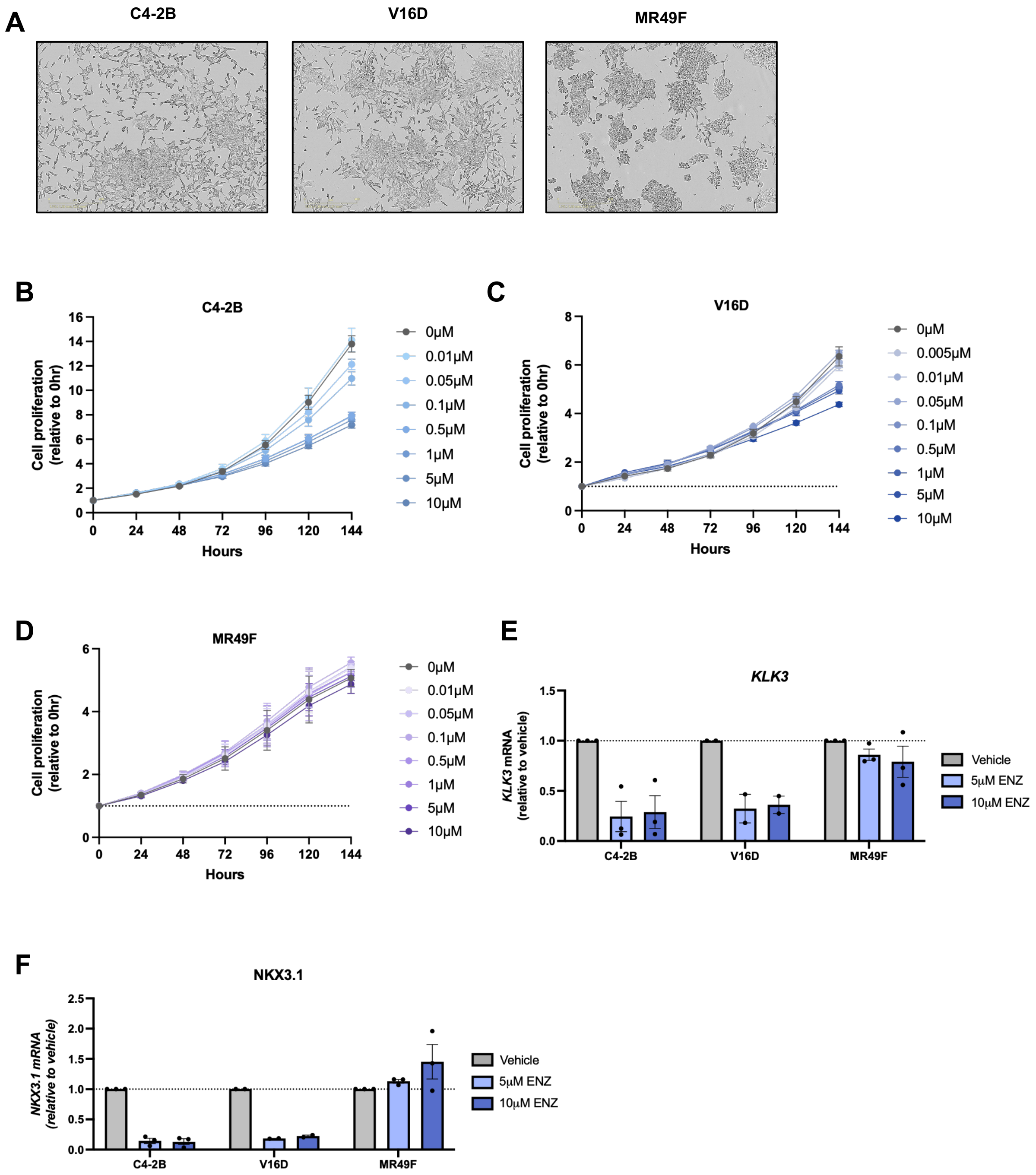

Supplementary Figure 1. Assessment of ENZ sensitivity of C4-2B, V16D and MR49F cells. **A** Phase images of C4-2B, V16D and MR49F cells imaged at 10X magnification IncuCyte SX5 live-cell imaging. **B** Cell proliferation of C4-2B cells treated with vehicle or ENZ (0.01, 0.05, 0.1, 0.5, 1, 5, 10 µM) over five days measured by IncuCyte SX5 live-cell imaging (n=3). **C** Cell proliferation of V16D cells treated with vehicle or ENZ (0.01, 0.05, 0.1, 0.5, 1, 5, 10 µM) over five days measured by IncuCyte SX5 live-cell imaging (n=3). **D** Cell proliferation of MR49F cells treated with vehicle or ENZ (0.01, 0.05, 0.1, 0.5, 1, 5, 10 µM) over five days measured by IncuCyte SX5 live-cell imaging (n=2). **E** *KLK3* mRNA expression in C4-2B and MR49F cells treated with vehicle or ENZ (5 µM, 10 µM) for 48 hours, measured by qRT-PCR analysis (n=3). **F** *NKX3.1* mRNA expression in C4-2B and MR49F cells treated with vehicle or ENZ (5 µM, 10 µM) for 48 hours, measured by qRT-PCR analysis (n=3). Statistical significance was determined using a one-way ANOVA or unpaired Welch's t-test, as described in the Methods. * p value < 0.05. Data are presented as mean ± SEM.

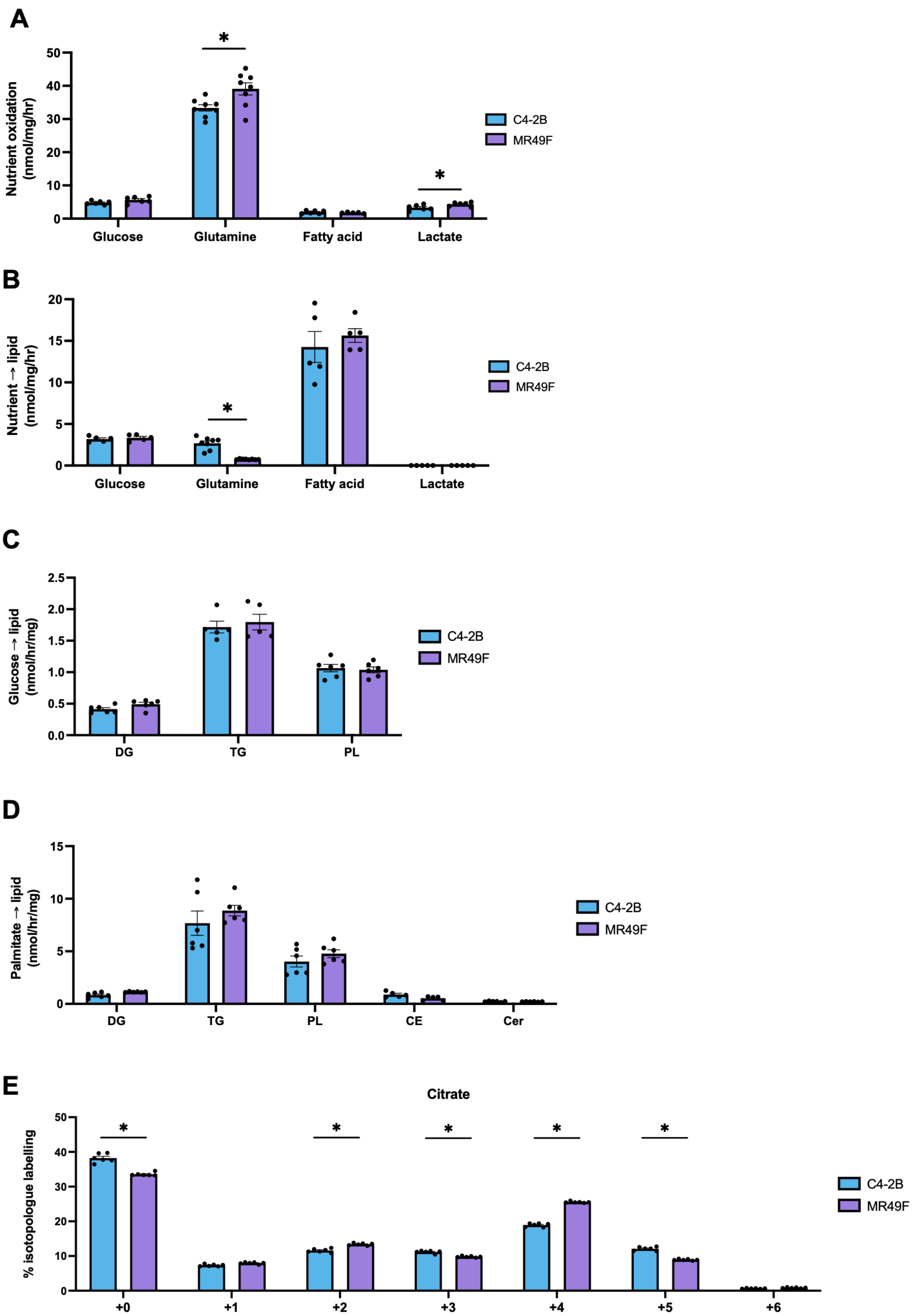
A
Nutrient oxidation (nmol/mg/hr)
50
40
30
20
10
0
*
*
Glucose
Glutamine
Fatty acid
Lactate
C4-2B
MR49F
B
Nutrient → lipid (nmol/mg/hr)
20
15
10
5
0
*
Glucose
Glutamine
Fatty acid
Lactate
C4-2B
MR49F
C
Glucose → lipid (nmol/hr/mg)
2.5
2.0
1.5
1.0
0.5
0.0
DG
TG
PL
C4-2B
MR49F
D
Palmitate → lipid (nmol/hr/mg)
15
10
5
0
DG
TG
PL
CE
Cer
C4-2B
MR49F
E
Citrate
% isotopologue labelling
50
40
30
20
10
0
*
*
*
*
*
+0
+1
+2
+3
+4
+5
+6
C4-2B
MR49F

Supplementary Figure 2. Nutrient consumption and utilisation in ENZ-sensitive and ENZ-resistant prostate cancer cells. **A** Rate of oxidation of glucose, glutamine, palmitate, and lactate in C4-2B and MR49F cells (nmol/mg/hr). **B** Rate of incorporation into lipid for glucose, glutamine, palmitate, and lactate in C4-2B and MR49F cells (nmol/mg/hr). **C** Rate of glucose incorporation into lipid classes (nmol/hr/mg) in C4-2B and MR49F cells. **D** Rate of palmitate incorporation into lipid classes (nmol/hr/mg) in C4-2B and MR49F cells. **E** Percent labelling of mass isotopologues of citrate with ^{13}C-glutamine in C4-2B and MR49F cells (n = 6). Abbreviations: triglyceride (TG), diglyceride (DG), phospholipid (PL), cholesterol ester (CE) and ceramide (Cer). Statistical significance was determined using a one-way ANOVA or unpaired Welch's t-test, as described in the Methods. * p value < 0.05. Data are presented as mean ± SEM.

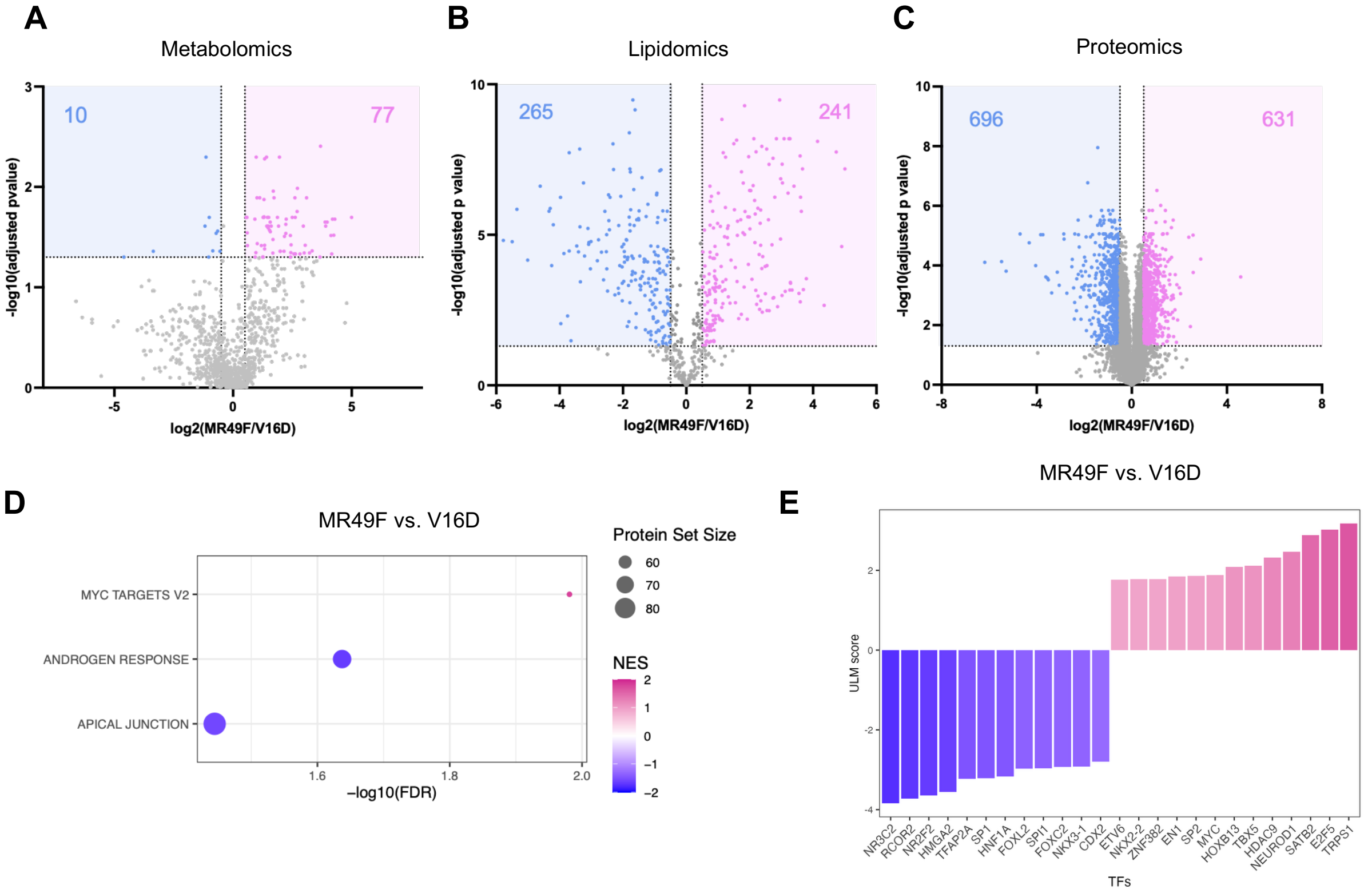

Supplementary Figure 3. Multi-omics analysis of MR49F cells relative to V16D cells. **A** Volcano plot of log2(fold change) expression of metabolites against -log10(adjusted p-value) comparing MR49F cells and V16D cells. **C** Volcano plot of log2(fold change) expression of lipids against -log10(adjusted p-value) comparing MR49F cells and V16D cells. **D** Volcano plot of log2(fold change) expression of proteins against -log10(adjusted p-value) comparing MR49F cells and V16D cells. **D** Protein set enrichment analysis (PSEA) of V16D and MR49F cells using HALLMARK, KEGG, and selected curated gene sets. **E** Transcription factor (TF) activity was inferred from proteomic differential expression data using the ULM method. Bars represent inferred TF activity scores, with colour indicating direction and magnitude of activity. The top 25 ranked TFs (p value < 0.05) are shown. Statistical significance was determined by unpaired two-tailed Welch's t-test with multiple testing correction, as described in the Methods.

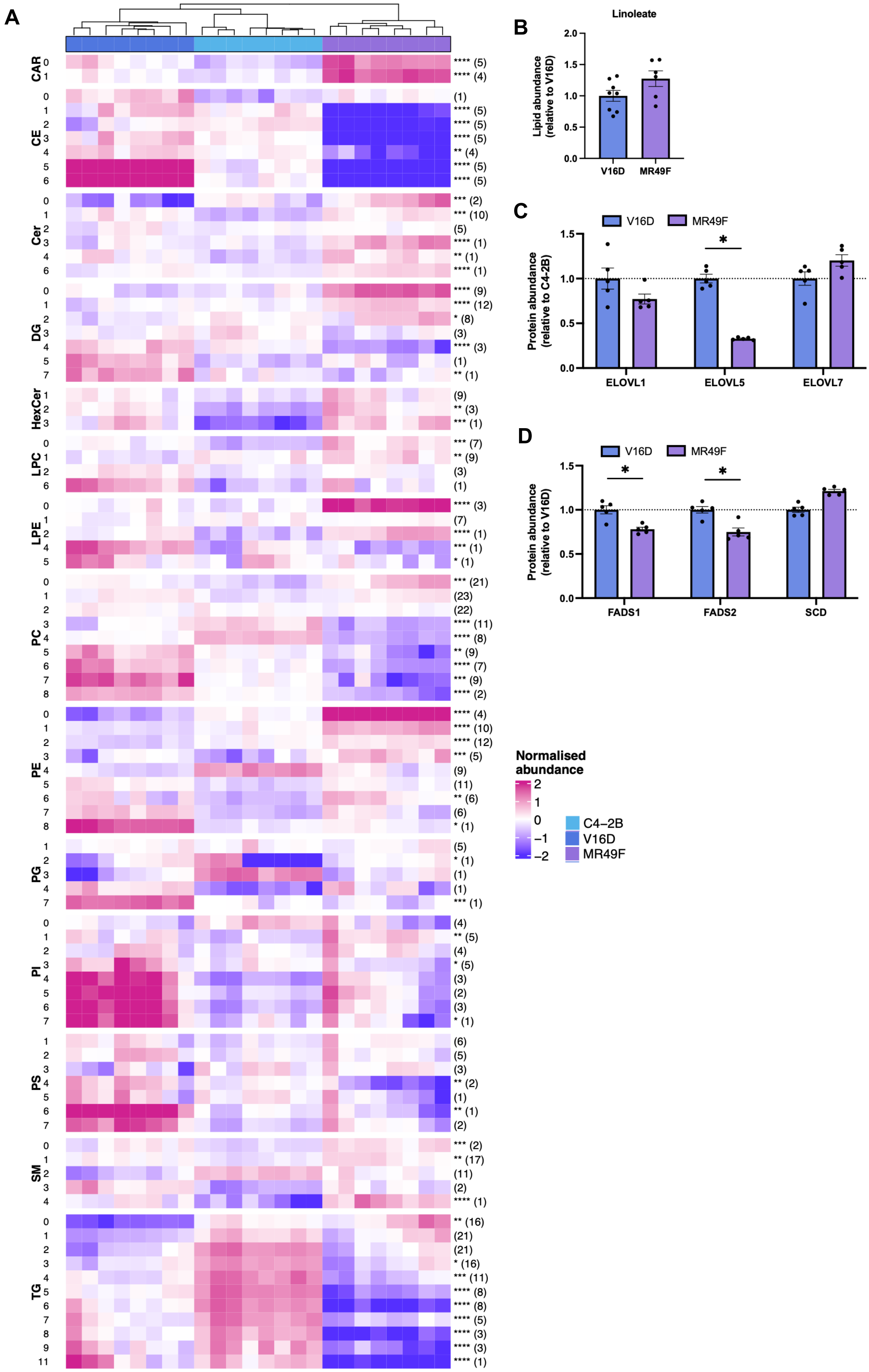

A
CAR
CE
Cer
DG
HexCer
LPC
LPE
PC
PE
PG
PI
PS
SM
TG
Normalised abundance
2
1
0
−1
−2
C4−2B
V16D
MR49F
B
Linoleate
Lipid abundance (relative to V16D)
V16D
MR49F
C
V16D
MR49F
Protein abundance (relative to C4-2B)
ELOVL1
ELOVL5
ELOVL7
D
V16D
MR49F
Protein abundance (relative to V16D)
FADS1
FADS2
SCD

Supplementary Figure 4. Reduced polyunsaturated fatty acid (PUFA) abundance across lipid classes in ENZ-resistant prostate cancer cells. **A** Heatmap of lipid abundance according to the number of carbons present in fatty acid chains grouped into individual lipid classes in C4-2B and MR49F cells. Individual samples were arranged by unsupervised clustering. **B** Linoleate (FA 18:2) abundance in V16D and MR49F cells (n=8) assessed by lipidomic analysis. **C** Protein content of elongase enzymes ELOVL1, ELOVL5 and ELOVL7 in V16D and MR49F cells (n=5). **D** Protein content of desaturase enzymes SCD, FADS1 and FADS2 in V16D and MR49F cells (n=5). Statistical significance was determined using a one-way ANOVA or unpaired Welch's t-test, as described in the Methods. * p value < 0.05. Data are presented as mean ± SEM.

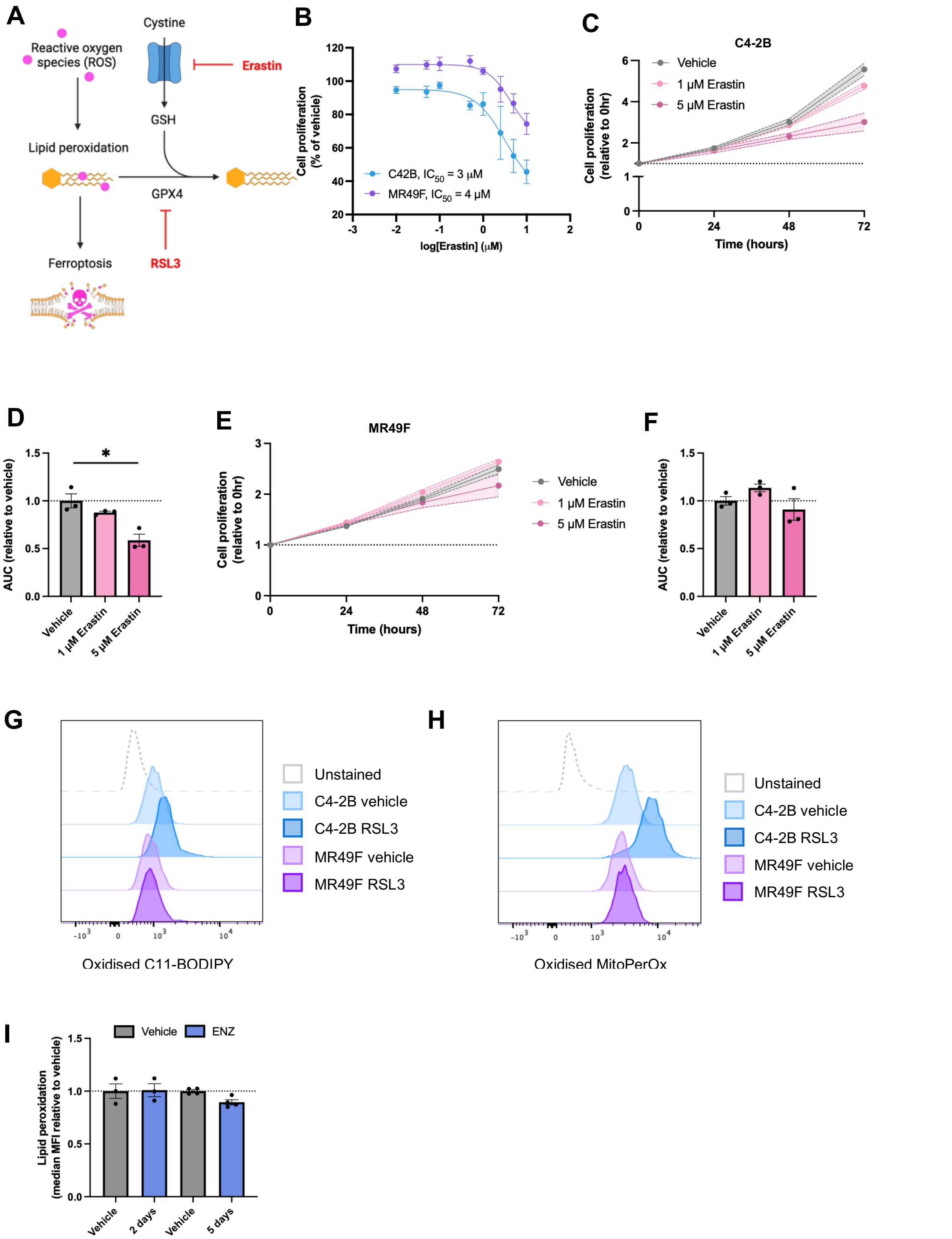

Supplementary Figure 5. ENZ-resistant prostate cancer cells are more resistant to RSL3 and erastin treatment. **A** Schematic illustrating process of glutathione dependent GPX4 and the uptake of cystine for glutathione synthesis by SLC7A11. Erastin is an inhibitor of SLC7A11.

Created in BioRender.com. **B** Dose-response curve analysis showing cell proliferation of C4-2B and MR49F cells treated with increasing doses of erastin for 72 hours. **C** Growth curves of C4-2B cells treating with vehicle or erastin (1 µM and 5 µM) over 72 hours, measured by IncuCyte SX5 live-cell imaging. **D** Area under curve (AUC, incremental) analysis of growth curves in C. **E** Growth curves of MR49F cells treating with vehicle or erastin (1 µM and 5 µM) over 72 hours, measured by IncuCyte SX5 live-cell imaging. **F** Area under curve (AUC, incremental) analysis of growth curves in E. **G** Representative histograms of C11-BODIPY intensity in C4-2B and MR49F cells treated with vehicle or RSL3 (2.5 µM) for 2 hours assessed by flow cytometry. **H** Representative histograms of MitoPerOx intensity in C4-2B and MR49F cells treated with vehicle or RSL3 (2.5 µM) for 2 hours assessed by flow cytometry. **I** Lipid peroxidation levels in C4-2B treated with ENZ for two and five days assessed by flow cytometry (C11-BODIPY, 2.5 µM) (n=3). Statistical significance was determined using a one-way ANOVA or unpaired Welch's t-test, as described in the Methods. * p value < 0.05. Data are presented as mean ± SEM.

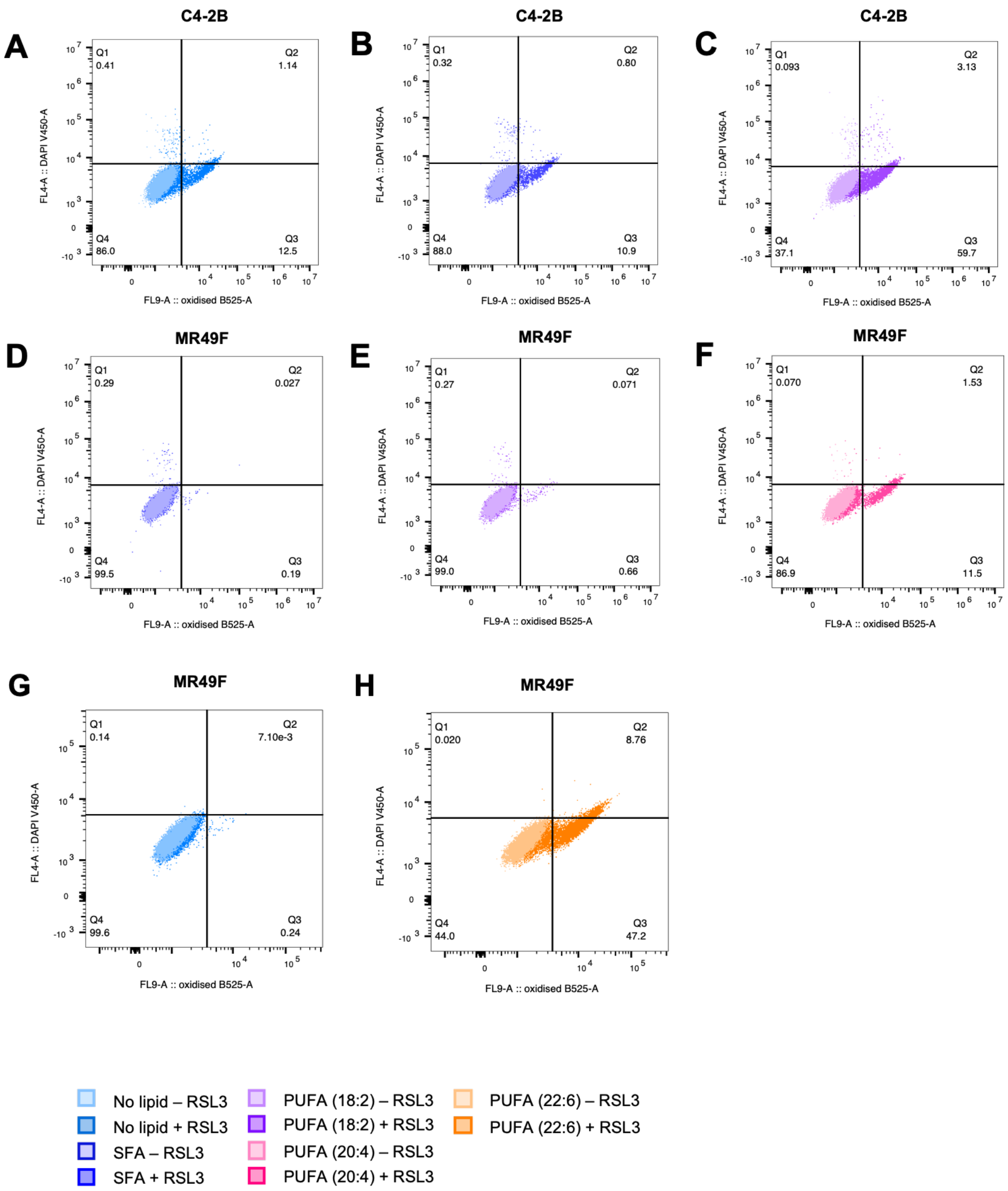

Supplementary Figure 6. PUFA supplementation does not affect the sensitivity of ENZ-sensitive cells to ferroptosis. **A** Representative dot plot of oxidised C11-BODIPY (B525) and DAPI (V450) of C4-2B cells treated with vehicle or RSL3 (2.5 µM, 2 hours). **B** Representative dot plot of oxidised C11-BODIPY (B525) and DAPI (V450) of C4-2B cells pre-treated with palmitic acid (SFA (18:0), 10 µM, 16 hours), and treated vehicle or RSL3 (2.5 µM, 2 hours). **C** Representative dot plot of oxidised C11-BODIPY (B525) and DAPI (V450) of C4-2B cells pre-treated with linoleic acid (PUFA (18:2), 10 µM, 16 hours), and

treated vehicle or RSL3 (2.5 µM, 2 hours). **D** Representative dot plot of oxidised C11-BODIPY (B525) and DAPI (V450) of MR49F cells pre-treated with palmitic acid (SFA (18:0), 10 µM, 16 hours), and treated vehicle or RSL3 (2.5 µM, 2 hours). **E** Representative dot plot of oxidised C11-BODIPY (B525) and DAPI (V450) of MR49F cells pre-treated with linoleic acid (PUFA (18:2), 10 µM, 16 hours), and treated vehicle or RSL3 (2.5 µM, 2 hours). **F** Representative dot plot of oxidised C11-BODIPY (B525) and DAPI (V450) of MR49F cells pre-treated with arachidonic acid (PUFA (20:4), 10 µM, 16 hours), and treated vehicle or RSL3 (2.5 µM, 2 hours). **G** Representative dot plot of oxidised C11-BODIPY (B525) and DAPI (V450) of MR49F cells pre-treated with no lipid and treated vehicle or RSL3 (2.5 µM, 2 hours). **H** Representative dot plot of oxidised C11-BODIPY (B525) and DAPI (V450) of C4-2B cells pre-treated with docosahexaenoic acid (PUFA (22:6), 10 µM, 16 hours), and treated vehicle or RSL3 (2.5 µM, 2 hours).

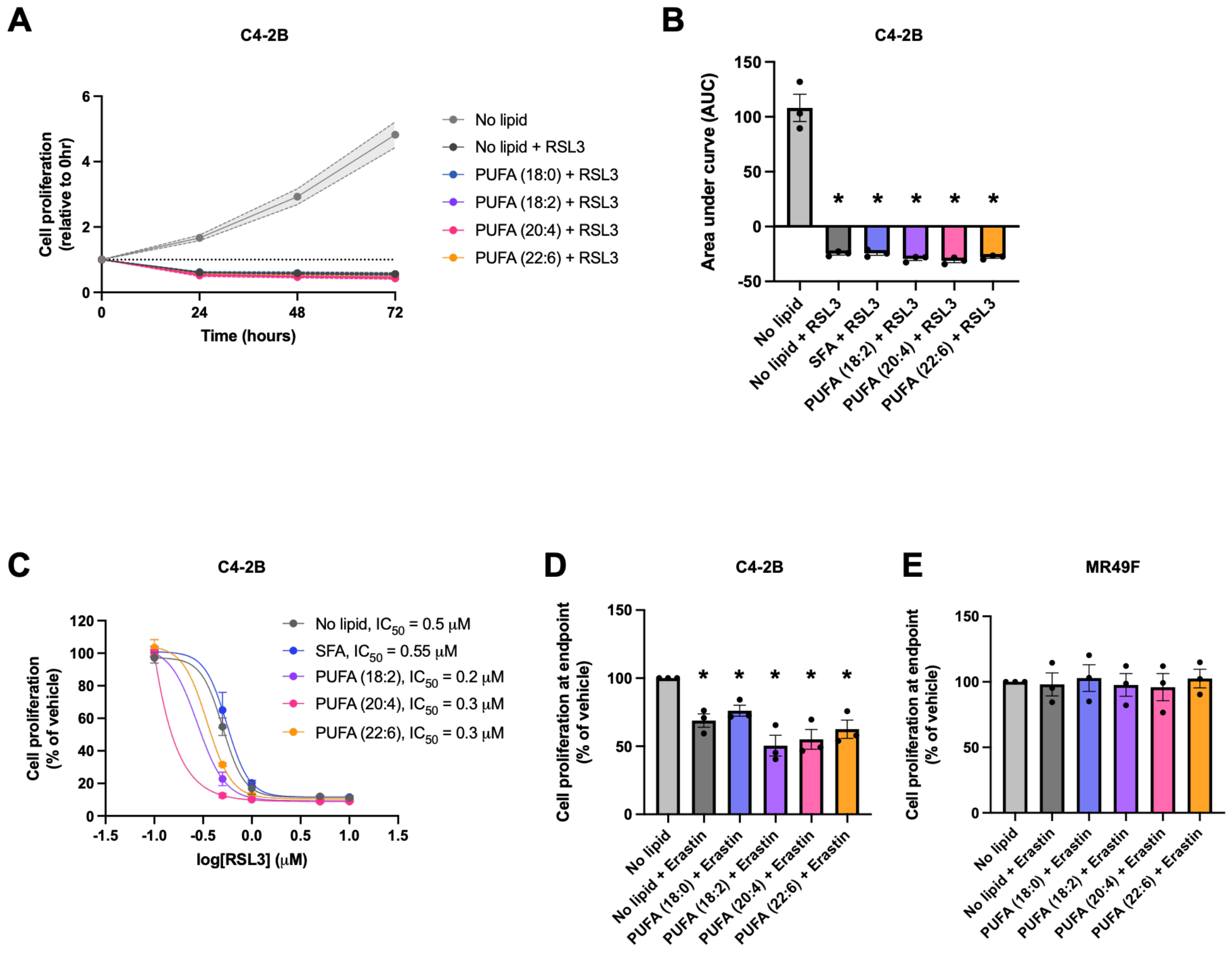

Supplementary Figure 7. PUFA supplementation and its effect on ferroptosis. **A** Dose-response curve analysis showing cell proliferation of C4-2B cells pre-treated with no lipid, palmitate (SFA, 10 µM, 16 hours), linoleate (PUFA (18:2), 10 µM, 16 hours), arachidonic acid (PUFA (20:4), 10 µM, 16 hours), or docosahexaenoic acid (PUFA (22:6), 10 µM, 16 hours), and treated with vehicle or RSL3 (0.1, 0.5, 1,5, and 10 µM) following 72 hours. **B** Growth curves of C4-2B cells pre-treated with no lipid, palmitate (SFA, 10 µM, 16 hours), linoleate (PUFA (18:2), 10 µM, 16 hours), arachidonic acid (PUFA (20:4), 10 µM, 16 hours), or docosahexaenoic acid (PUFA (22:6), 10 µM, 16 hours), and treated with vehicle or RSL3 (5 µM) over 72 hours, measured by IncuCyte SX5 live-cell imaging. **C** Area under curve (AUC, incremental) analysis of growth curves in B. **D** Cell proliferation of C4-2B cells pre-treated with no lipid, palmitate (SFA, 10 µM, 16 hours), linoleate (PUFA (18:2), 10 µM, 16

hours), arachidonic acid (PUFA (20:4), 10 µM, 16 hours), or docosahexaenoic acid (PUFA (22:6), 10 µM, 16 hours), and treated with vehicle or erastin (5 µM) following 72 hours. **E** Cell proliferation of MR49F cells pre-treated with no lipid, palmitate (SFA, 10 µM, 16 hours), linoleate (PUFA (18:2), 10 µM, 16 hours), arachidonic acid (PUFA (20:4), 10 µM, 16 hours), or docosahexaenoic acid (PUFA (22:6), 10 µM, 16 hours), and treated with vehicle or erastin (5 µM) following 72 hours. Statistical significance was determined using a one-way ANOVA or unpaired Welch's t-test, as described in the Methods. * p value < 0.05. Data are presented as mean ± SEM.

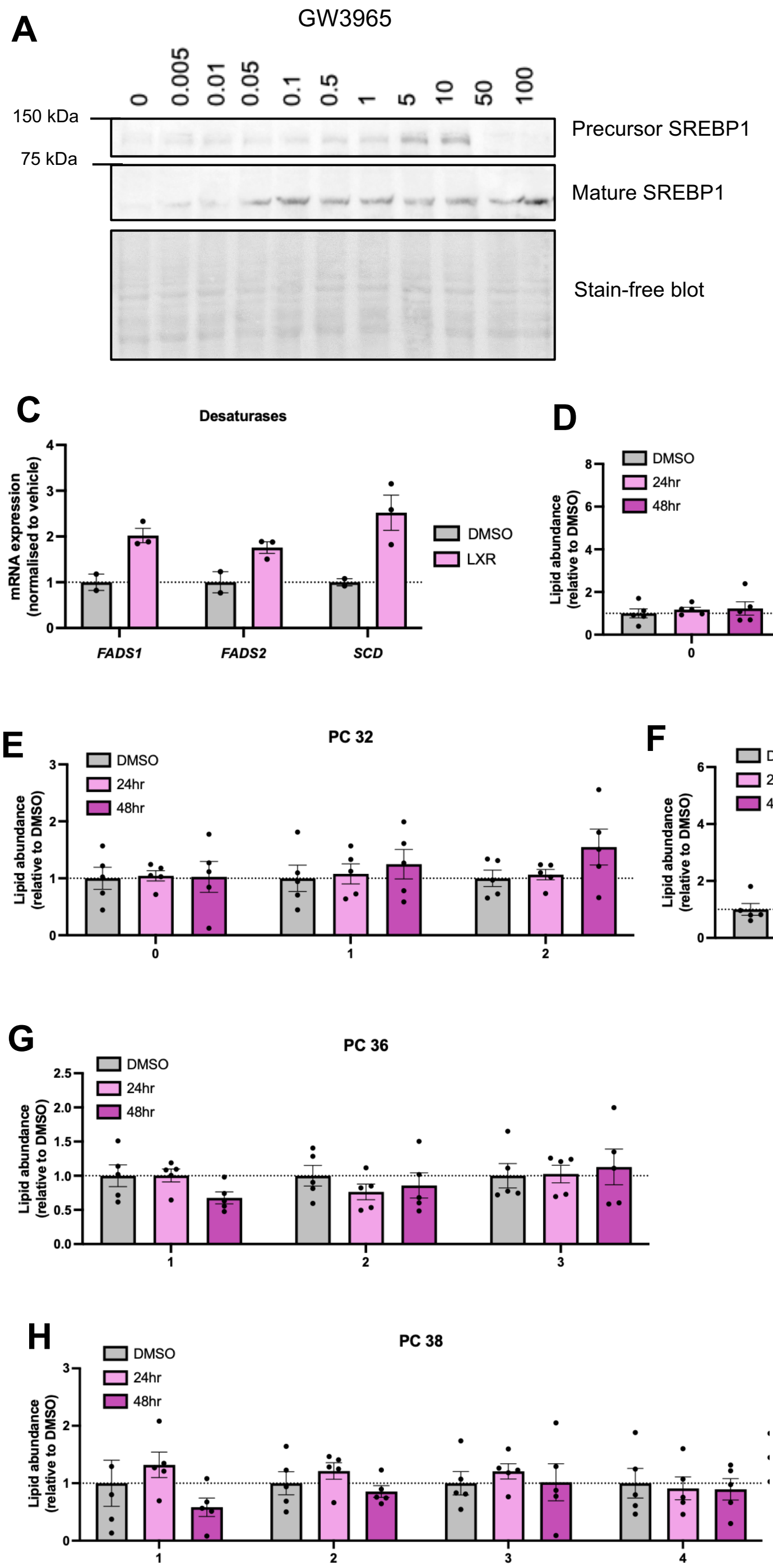
A
GW3965
0
0.005
0.01
0.05
0.1
0.5
1
5
10
50
100
150 kDa
75 kDa
Precursor SREBP1
Mature SREBP1
Stain-free blot
B
Elongases
mRNA expression (normalised to vehicle)
ELOVL5
ELOVL7
DMSO
LXR
C
Desaturases
FADS1
FADS2
SCD
D
PC 30
Lipid abundance (relative to DMSO)
DMSO
24hr
48hr
****
*
**
E
PC 32
F
PC 34
***
G
PC 36
H
PC 38

Supplementary Figure 8. Restoring mature SREBP1 levels with LXR agonist GW3965 re-sensitises ENZ-resistant cells to RSL3. **A** Immunoblot (cropped) of precursor and mature SREBP1 in MR49F cells treating with increasing concentrations of GW3965 (0, 0.005, 0.01, 0.05, 0.1, 0.5, 1, 5, 10, 50, 100 µM) for 16 hours. **B** Elongase (ELOVL5 and ELOVL7) mRNA expression in MR49F cells treated with vehicle or GW3965 (0.5 µM, 24 h). **C** Desaturase (FADS1, FADS2 and SCD) mRNA expression in MR49F cells treated with vehicle or GW3965 (0.5 µM, 24 h). **D** Lipid abundance of PC 30 with 0, 1 or 2 double bonds in MR49F cells treated with vehicle or GW3965 for 24 and 48 h. **E** Lipid abundance of PC 32 with 0, 1, or 2 double bonds in MR49F cells treated with vehicle or GW3965 for 24 and 48 h. **F** Lipid abundance of PC 34 with 1, 2, or 3 double bonds in MR49F cells treated with vehicle or GW3965 for 24 and 48 h. **G** Lipid abundance of PC 36 with 1, 2, or 3 double bonds in MR49F cells treated with vehicle or GW3965 for 24 and 48 h. **H** Lipid abundance of PC 38 with 1, 2, 3 or 4 double bonds in MR49F cells treated with vehicle or GW3965 for 24 and 48 h. Statistical significance was determined using a one-way ANOVA or unpaired Welch's t-test, as described in the Methods. * p value < 0.05. Data are presented as mean ± SEM.

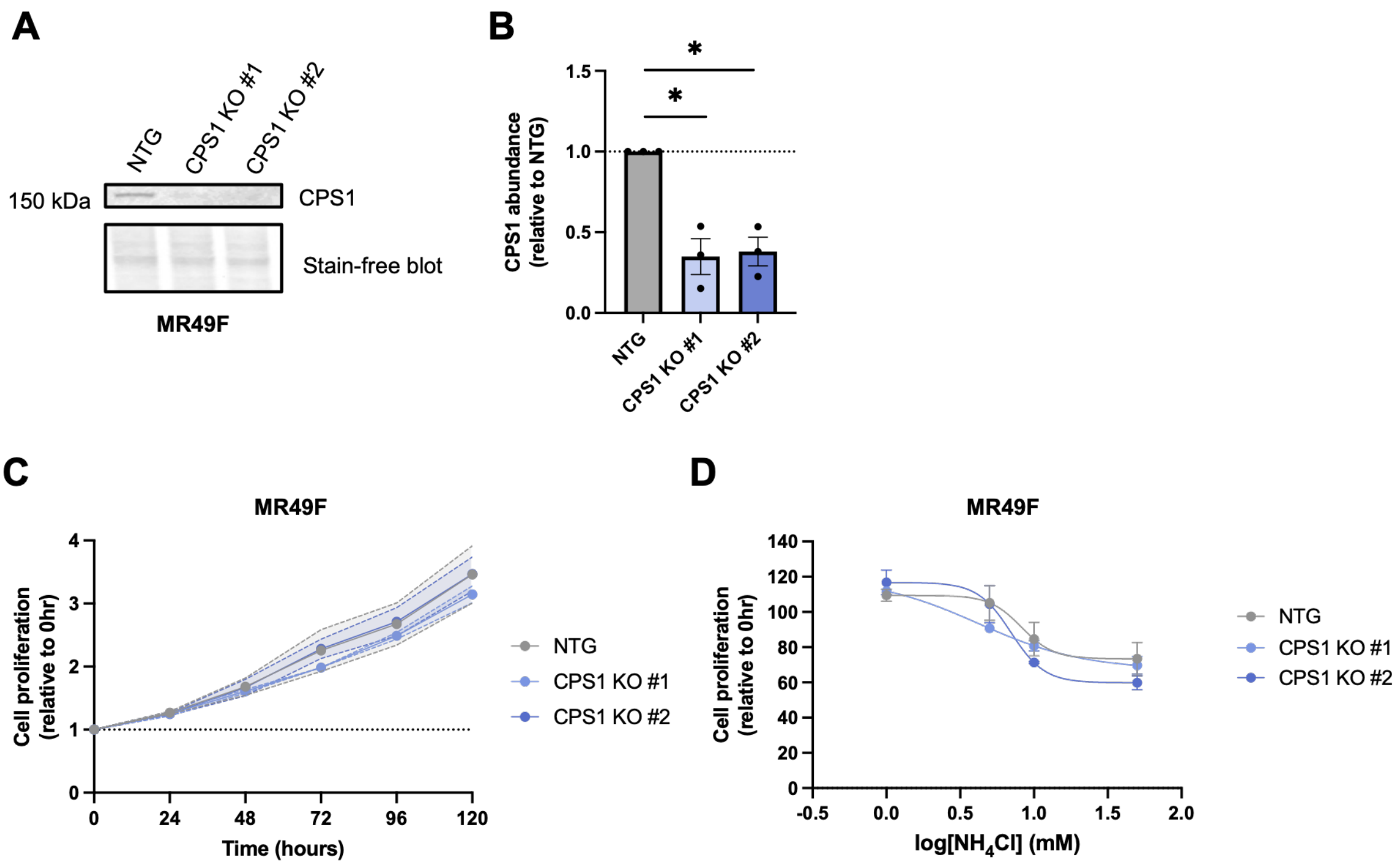

Supplementary Figure 9. Genetic ablation of CPS1 does not affect ENZ-resistant prostate cancer cell proliferation nor sensitivity to ammonium exposure. **A** Immunoblot (cropped) of CPS1 in MR49F cells transduced with a control guide (NTG) or guide targeting CPS1 (CPS1 KO #1, CPS1 KO #2) five days following doxycycline treatment. **B** Quantification of immunoblot in A. **C** Cell proliferation of NTG, CPS1 KO #1, and CPS1 KO #2 MR49F cells transduced with a control guide (NTG) or guide targeting CPS1 (CPS1 KO #1, CPS1 KO #2) five days following doxycycline treatment over five days measured by IncuCyte SX5 live-cell imaging. **D** Dose-response curve (non-linear fit) showing percentage cell proliferation of NTG, CPS1 KO #1, and CPS1 KO #2 MR49F cells at increasing concentrations of NH_4Cl (1, 5, 10, 50 mM) following five days of treatment. Statistical significance was determined using a one-way ANOVA or unpaired Welch's t-test, as described in the Methods. * p value < 0.05. Data are presented as mean ± SEM.

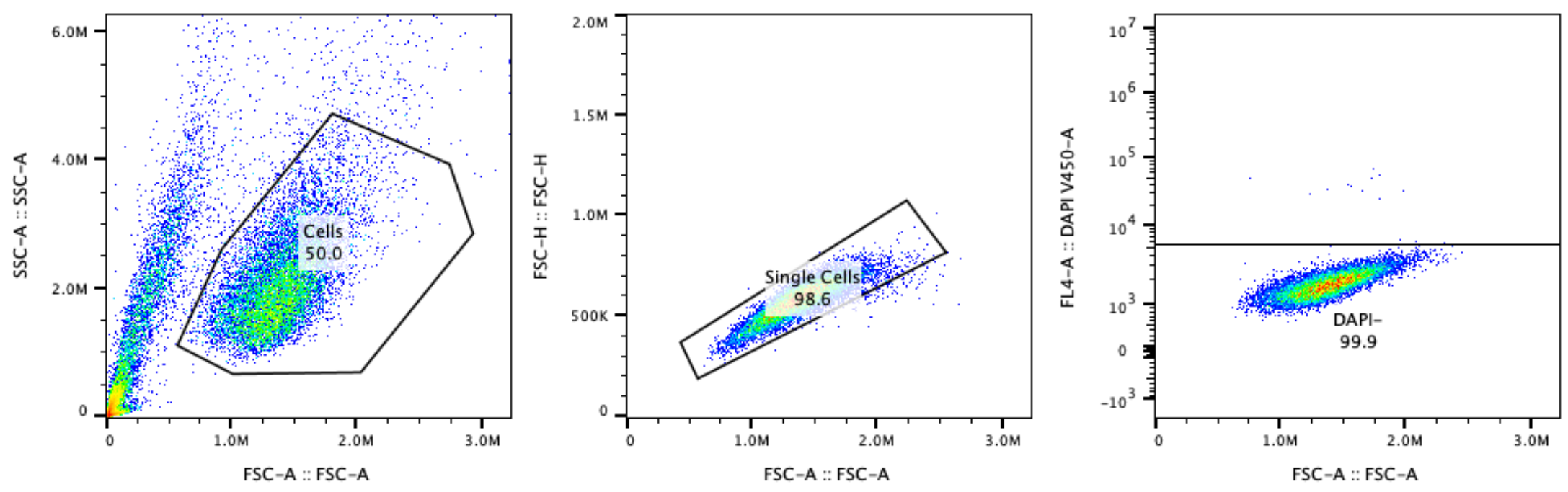

Supplementary Figure 10. Gating strategy for flow cytometry analysis.

Omics analysis	Features after QC	Detected in >80% samples	Common	Only detected in C4-2B	Only detected in V16D	Only detected in MR49F
Lipidomics	762	751	578	0	0	0
Metabolomics	1163	1162	1162	0	0	0
Proteomics	7993	7626	7364	59	44	19

Supplementary Table 1. Feature detection and coverage across multi-omic datasets.

Gene	Forward primer sequence (5’-3’)	Reverse primer sequence (5’-3’)
KLK3	CACACCCGCTCTACGATATG	GAGGTCCACACACTGAAGTT
NKX3.1	GGCCTGGGAGTCTCTTGACTCCACTAC	ATGTGGAGCCCAAACCACAGAAAATG
CPS1	ATTCCTTGGTGTGGCTGAAC	ATGGAAGAGAGGCTGGGATT
ELOVL5	TTCATCCTGCGCAAGAACAACCAC	ATGGAAGGGACTGACGACAAACCA
ELOVL7	TCTATGAATCCTTGAGGGCCTA	TGACAACATCCACAGAATGTTCC
FADS1	CCATGCCAAGCCCAACTGCT	TGTGCTGGTGGTTGTACGGC
FADS2	AGCACCACGCCAAGCCTAAC	GCTGCCATTCGCCCAGAACAA
SCD	TCTAGCTCCTATACCACCACCA	TCGTCTCCAACTTATCTCCTCC
RPLP0	CAGATTGGCTACCCAACTGTT	GGGAAGGTGTAATCCGTCTCC
ACTB	AGAGCTACGAGCTGCCTGAC	AGCACTGTGTTGGCGTACAG

Supplementary Table 2. Primer sequences used for qRT-PCR analysis.

Oligo pair	Top oligo (5’ - 3’)	Bottom oligo (5’ - 3’)
**NTG**	CGTATTACTGATATTG GTGGGGTTTA	CCCACCAATATCAGTA ATACGGTGTC
**CPS1 KO #1**	CGTTTTGATCGCGGGAGGAC CGGTTTA	CGGTCCTCCCGCGATCAAAA CGGTGTC
**CPS1 KO #2**	CGCATAGATACGCGTGCTGC TGTTTA	AGCAGCACGCGTATCTATGC GGTGTC

Supplementary Table 3. Oligo sequences used for generation of CPS1 KO lines.
