# Supplementary material for: Multi-omic profiling reveals metabolic vulnerabilities in enzalutamide resistant prostate cancer
Source: Cell Death Discov. 2026 Sep 9;12:431. doi: 10.1038/s41420-026-03332-3 (PMC13720084; doi:10.1038/s41420-026-03332-3)
Supplement: Supplementary file 2 [file 41420_2026_3332_MOESM3_ESM.pdf]

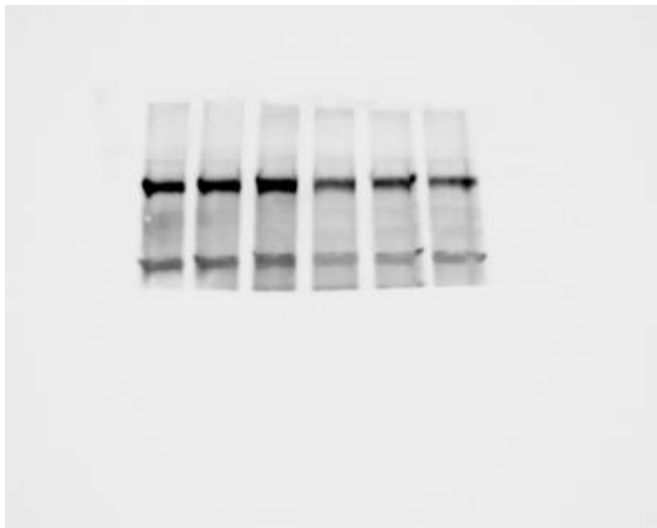
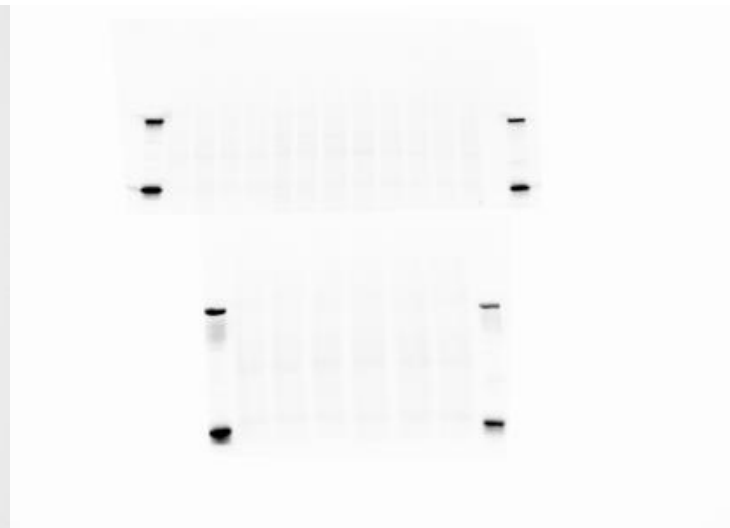
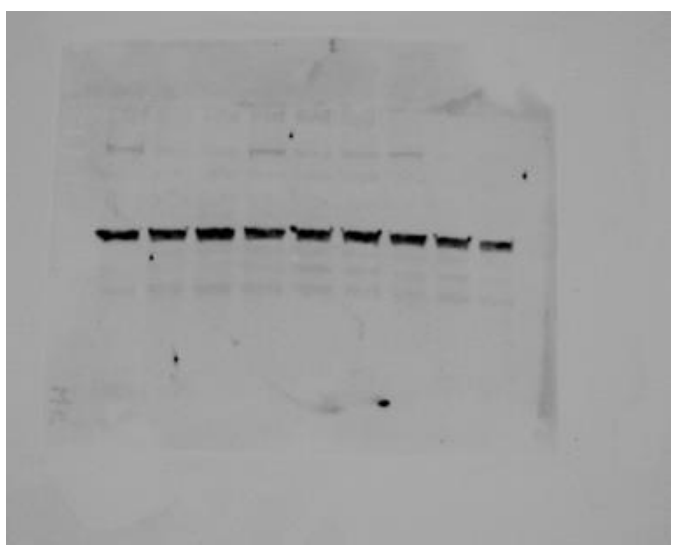
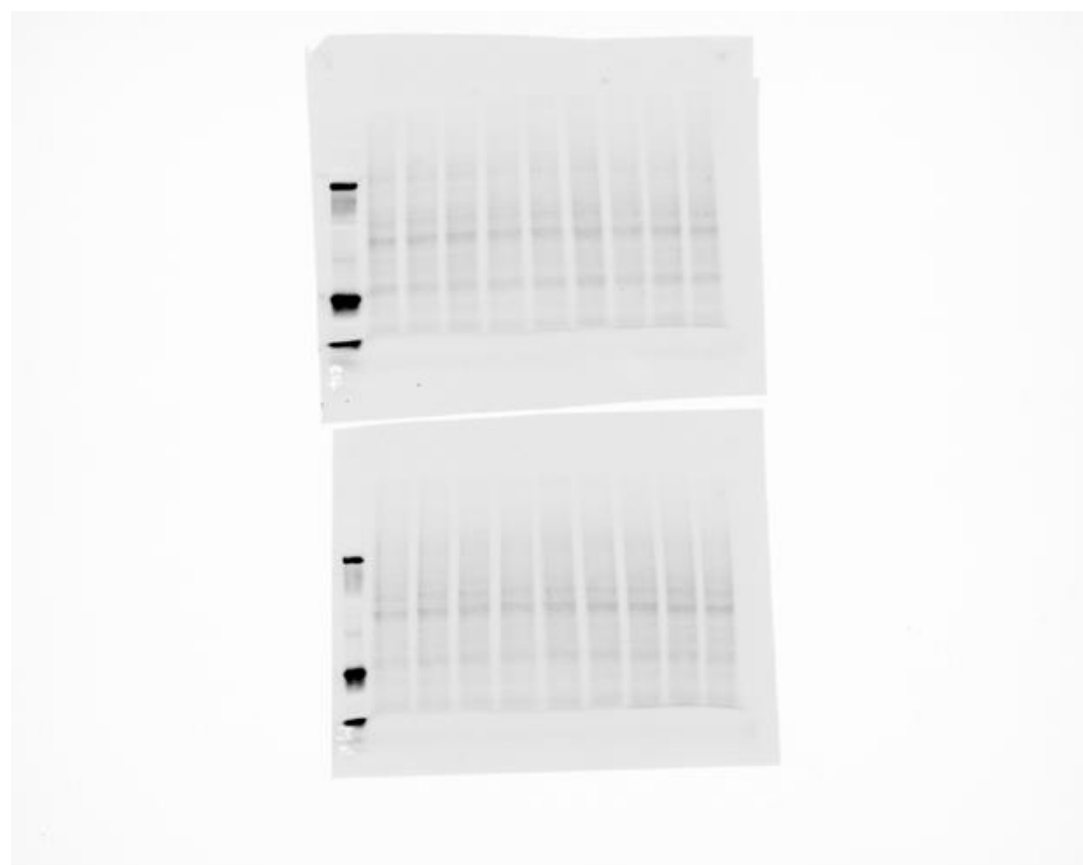
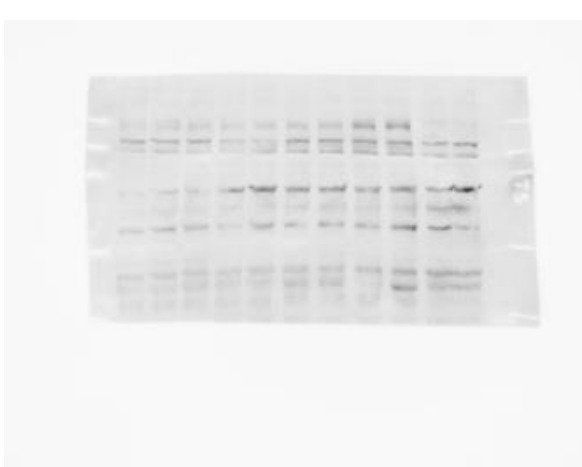
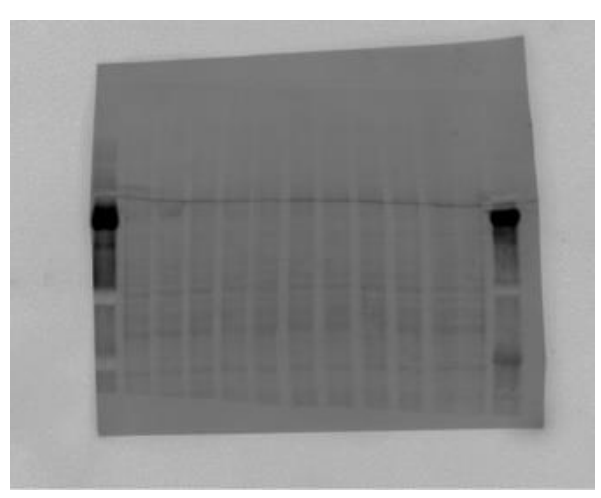
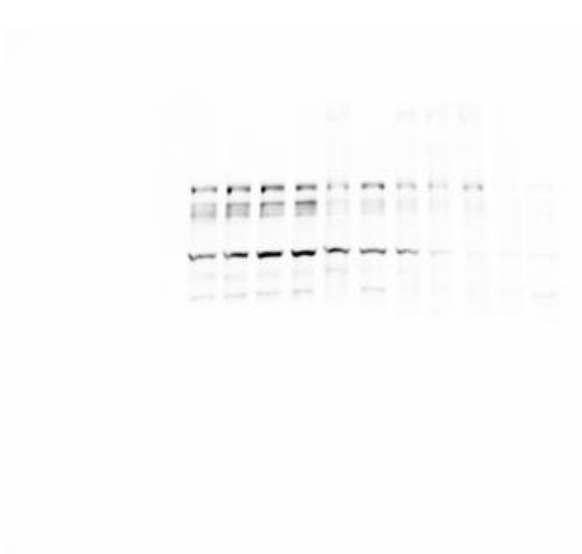
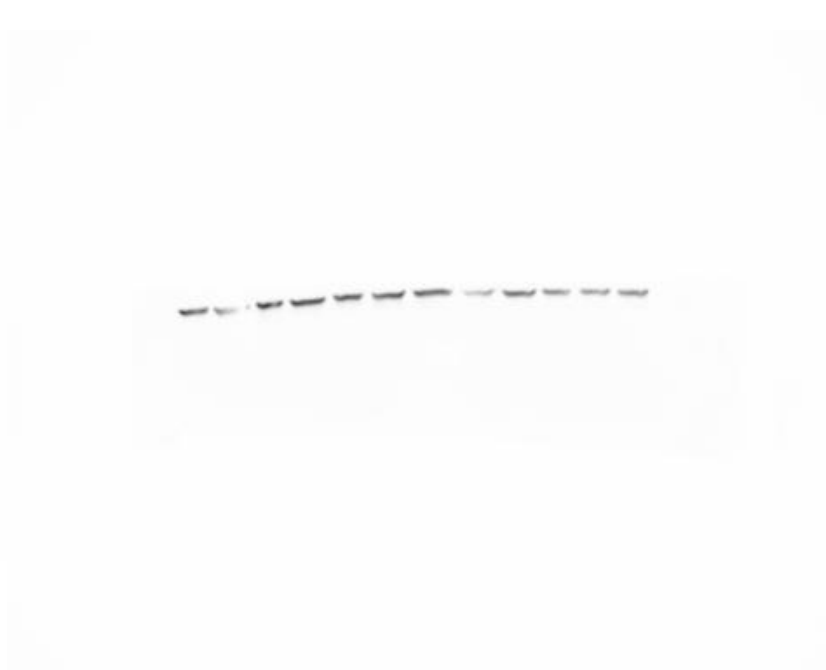

Original western blots
